# A Family of Related Fungal and Bacterial Di‐ and Sesterterpenes: Studies on Fusaterpenol and Variediene

**DOI:** 10.1002/cbic.201900462

**Published:** 2019-11-07

**Authors:** Jan Rinkel, Simon T. Steiner, Guangkai Bian, Rong Chen, Tiangang Liu, Jeroen S. Dickschat

**Affiliations:** ^1^ Kekulé Institute of Organic Chemistry and Biochemistry University of Bonn Gerhard-Domagk-Strasse 1 53121 Bonn Germany; ^2^ Key Laboratory of Combinatorial Biosynthesis and Drug Discovery Ministry of Education and Wuhan University School of Pharmaceutical Sciences 185 Dunghu Road Wuhan 430071 P. R. China

**Keywords:** absolute configuration, biosynthesis, isotopes, terpenes, thermal rearrangement

## Abstract

The absolute configuration of fusaterpenol (GJ1012E) has been revised by an enantioselective deuteration strategy. A bifunctional enzyme with a terpene synthase and a prenyltransferase domain from *Aspergillus brasiliensis* was characterised as variediene synthase, and the absolute configuration of its product was elucidated. The uniform absolute configurations of these and structurally related di‐ and sesterterpenes together with a common stereochemical course for the geminal methyl groups of GGPP unravel a similar conformational fold of the substrate in the active sites of the terpene synthases. For variediene, a thermal reaction observed during GC/MS analysis was studied in detail for which a surprising mechanism was uncovered.

## Graphical abstract

**Absolute configuration of fusaterpenol (GJ1012E) revised**: Variediene isolated from a newly characterised fungal diterpene synthase has a related absolute configuration. The comparison to other known diterpenes and labelling experiments point to a common substrate fold for a family of related compounds. A thermal reaction of variediene observed during GC/MS analysis is studied in detail.

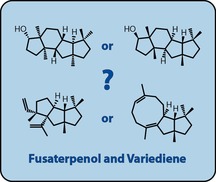

During the past few years, a series of structurally related di‐ and sesterterpenes from fungi and bacteria has been identified.^1^, ^2^ These compounds were either isolated from culture extracts, or obtained in genome‐mining approaches by heterologous gene expression in suitable hosts for compound production and isolation or by in vitro incubations of the terpene precursor with the purified enzyme. All these compounds have a *cis*‐fused cyclopentane ring that arises biosynthetically from geranylgeranyl diphosphate (GGPP) or geranylfarnesyl diphosphate (GFPP) by an initial 1,11–10,14 cyclisation to the cationic intermediate **A** and a subsequent ring expansion with simultaneous ring contraction to the hypothetical secondary cation **B** that can stabilise by further 2,10 or 6,10 cyclisation (Scheme 1 A). The known fungal representatives are generally made by bifunctional enzymes containing a terpene synthase domain and a prenyltransferase domain for GGPP or GFPP biosynthesis (TS+PT)^3^ and include phomopsene (**1**) and its derivative methyl phomopsenoate (**2**) from *Phomopsis amygdali*,^4^ variediene (**3**) from *Emericella variecolor* 5 and the cyclopiane‐type diterpene (**4**) from *Penicillium chrysogenum* (Scheme 1 B).^6^ Compound **4** is the biosynthetic precursor of a group of oxidised derivatives such as conidiogenone (**5**), one of the first isolated compounds of this class that exhibits an interesting bioactivity as a potent inducer of conidiogenesis in *Penicillium*.^7^

**Scheme 1:**
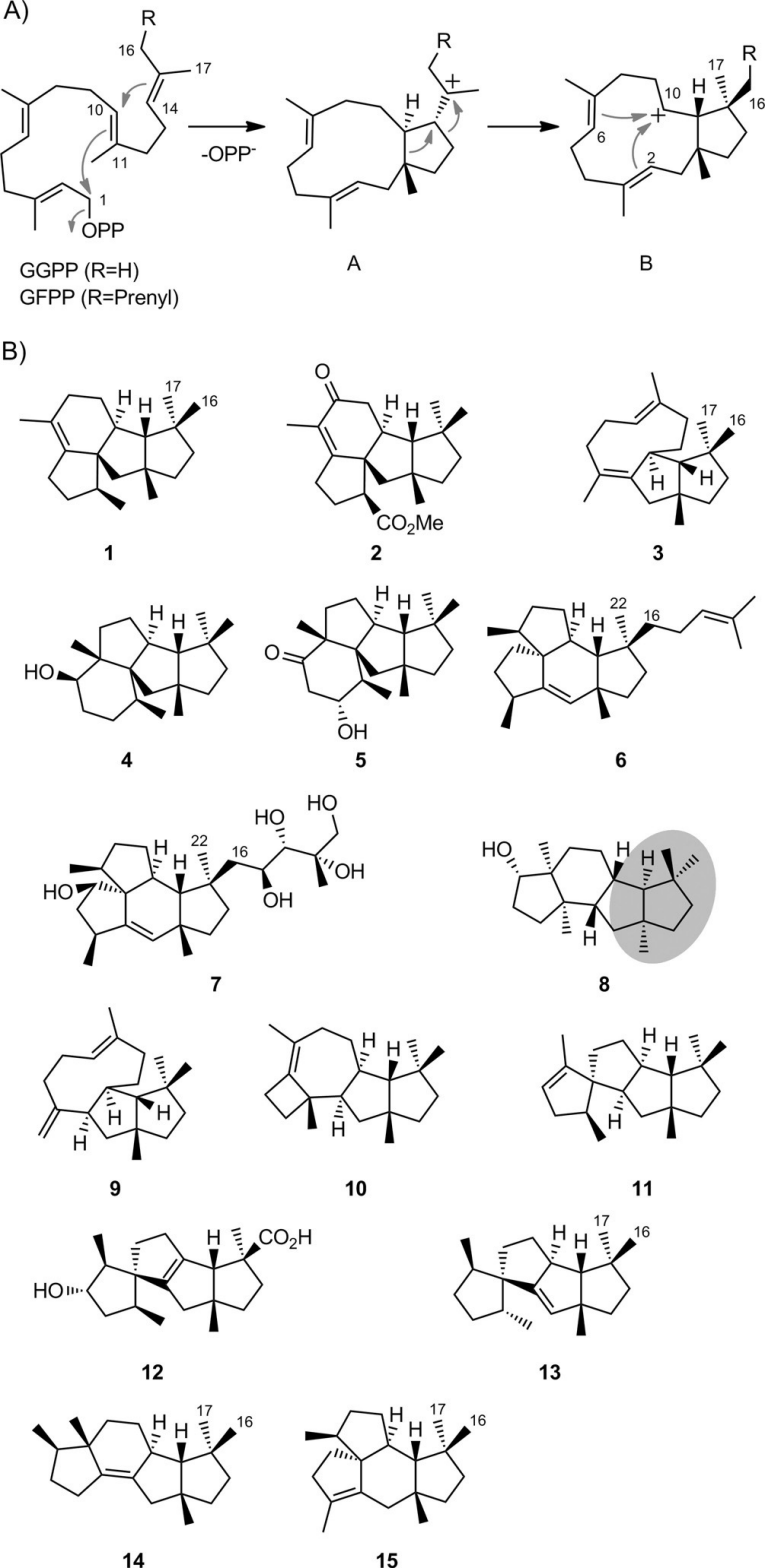
A) Initial cyclisation steps towards B) a group of related terpenoids from both fungal and bacterial sources. The fate of the geminal Me groups C16 and C17 of GGPP and of C16 and C22 of GFPP (C22 of GFPP equals C17 of GGPP) is indicated for all investigated cases.

Recently, a bifunctional sesterterpene synthase for mangicdiene (**6**) was identified from *Fusarium graminearum*.^8^ This compound is the likely biosynthetic precursor for mangicol A (**7**) and other mangicols found in a marine *Fusarium* isolate.^9^ In contrast, a monofunctional diterpene synthase from *F. graminearum* gave a mixture of compounds with the diterpene alcohol **8** as main product, a compound that was provisionally designated by us GJ1012E and for which we now propose the name fusaterpenol. Side products made by the fusaterpenol synthase FgGS include **3**, its double‐bond isomer **9** and the related compounds **10** and **11**.^8^ Spirocyclic **11** is structurally similar to spirograterpene A (**12**) from *Penicillium granulatum* 10 and to the bacterial compound spiroviolene (**13**) from *Streptomyces violens*, for which the diterpene synthase has recently been reported.^11^ Further recently characterised diterpene synthases include the cattleyene (**14**) synthase from *Streptomyces cattleya* 12 and the allokutznerene (**15**) synthase from *Allokutzneria albata*, which produces a mixture of **15** and **1**.^13^ Furthermore, phomopsene synthases are known from *Nocardia testacea* and *Nocardia rhamnosiphila* that convert GGPP into **1** as a single product.^12^

The absolute configurations of **2**, **3**, **7** and **12** 4, 5, 9, 10 were determined by modified Mosher's method,^14^ and the absolute configuration of fungal **1** was deduced from that of **2**.^4^ The absolute configurations of bacterial **1** and the other bacterial diterpene synthase products **13**–**15** were determined by chemical correlation using enantioselectively deuterated GGPP isotopomers.^11^, ^12^, ^13^ For compound **4**, the crystalline sponge method was applied,^6^ an X‐ray‐based technique that allows structural data to be determined from small quantities of material.^15^ The absolute configuration of its derivative **5** was based on an enantioselective total synthesis,^16^ whereas for **6** and **9**–**11** obtained from *Fusarium* enzymes, calculated and experimental electronic circular dichroism (ECD) spectra were compared. Compound **3** from *Fusarium* was shown to have the same absolute configuration as **3** from *E. variecolor* based on a comparison of their optical rotations. Finally, the absolute configuration of **8** was obtained from crystal structure data, but was based on a fairly poor Flack parameter (−0.1(5)).^8^ Notably, the stereochemistry at the initially formed cyclopentane ring of all these molecules is the same, only for compound **8** is a different stereochemistry found (grey shaded area in Scheme 1 B); more importantly, this stereochemistry does not fit the configurations of the other products **3** and **9**–**11** of FgGS. Herein, we report a reinvestigation of the absolute configuration of **8** and mechanistic studies with FgGS. Furthermore, a newly characterised variediene synthase from *Aspergillus brasiliensis* is reported, and its stereochemical course is compared to that of the known related bacterial and fungal enzymes.

As pointed out, the formation of all diterpenes shown in Scheme 1, apart from **8**, can be explained by initial cyclisation reactions to cation **A**, whereas the opposite absolute configuration of *ent*‐**8** should be expected if this compound also arose via **A** (Scheme 2 A). To reinvestigate the absolute configuration of **8**, its NMR data in C_6_D_6_ were analysed (Table S1 and Figures S1–S8 in the Supporting Information); NOE correlations yielded the relative orientation of all methylene groups in **8**. In an isotopic‐labelling approach, dimethylallyl diphosphate (DMAPP) and (*E*)‐ and (*Z*)‐(4‐^13^C,4‐^2^H)IPP^13^ (IPP=isopentenyl diphosphate) were enzymatically converted with farnesyl diphosphate synthase (FPPS) from *Streptomyces coelicolor* 17 and GGPP synthase (GGPPS) from *Streptomyces cyaneofuscatus* 11 into isotopically substituted GGPP with enantioselective deuterations at C4, C8 and C12 (Scheme 2 B). Subsequent cyclisation with FgGS yielded products in which the relative orientations of the incorporated deuterium atoms were in agreement with the structure of *ent*‐**8**, but not of **8**. The additional ^13^C labels in this experiment served for the highly sensitive analysis of the obtained products by HSQC spectroscopy (Figure 1 A), without the need for compound purification, so that these labelling experiments could be performed with small quantities of labelled material.

**Scheme 2:**
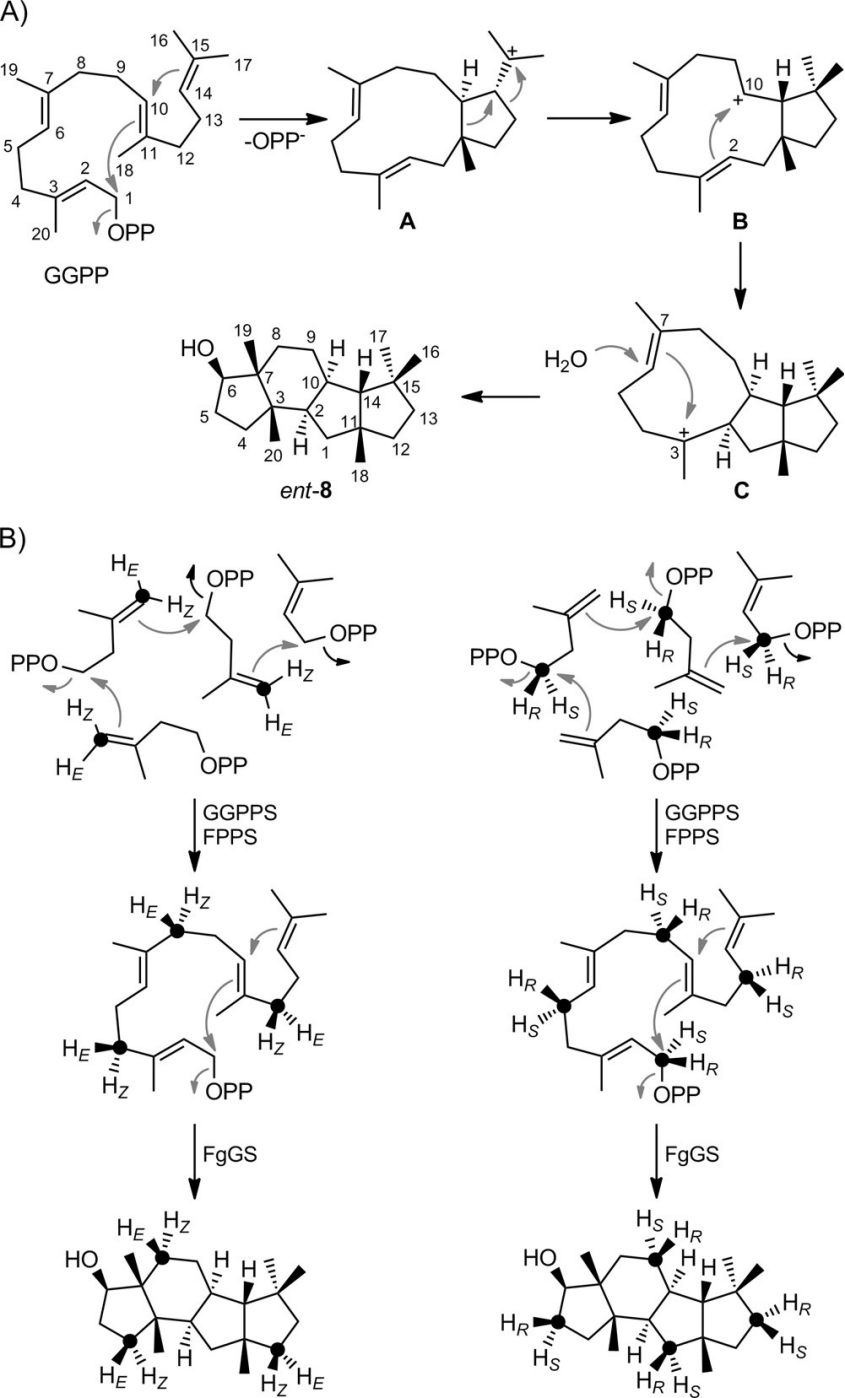
A) Proposed cyclisation mechanism to *ent*‐**8** and B) conversion of selectively labelled IPP isotopomers to labelled *ent*‐**8** concluding on its absolute configuration (cf. Figure 1). Black dots represent ^13^C‐labelled carbon atoms.

**Figure 1:**
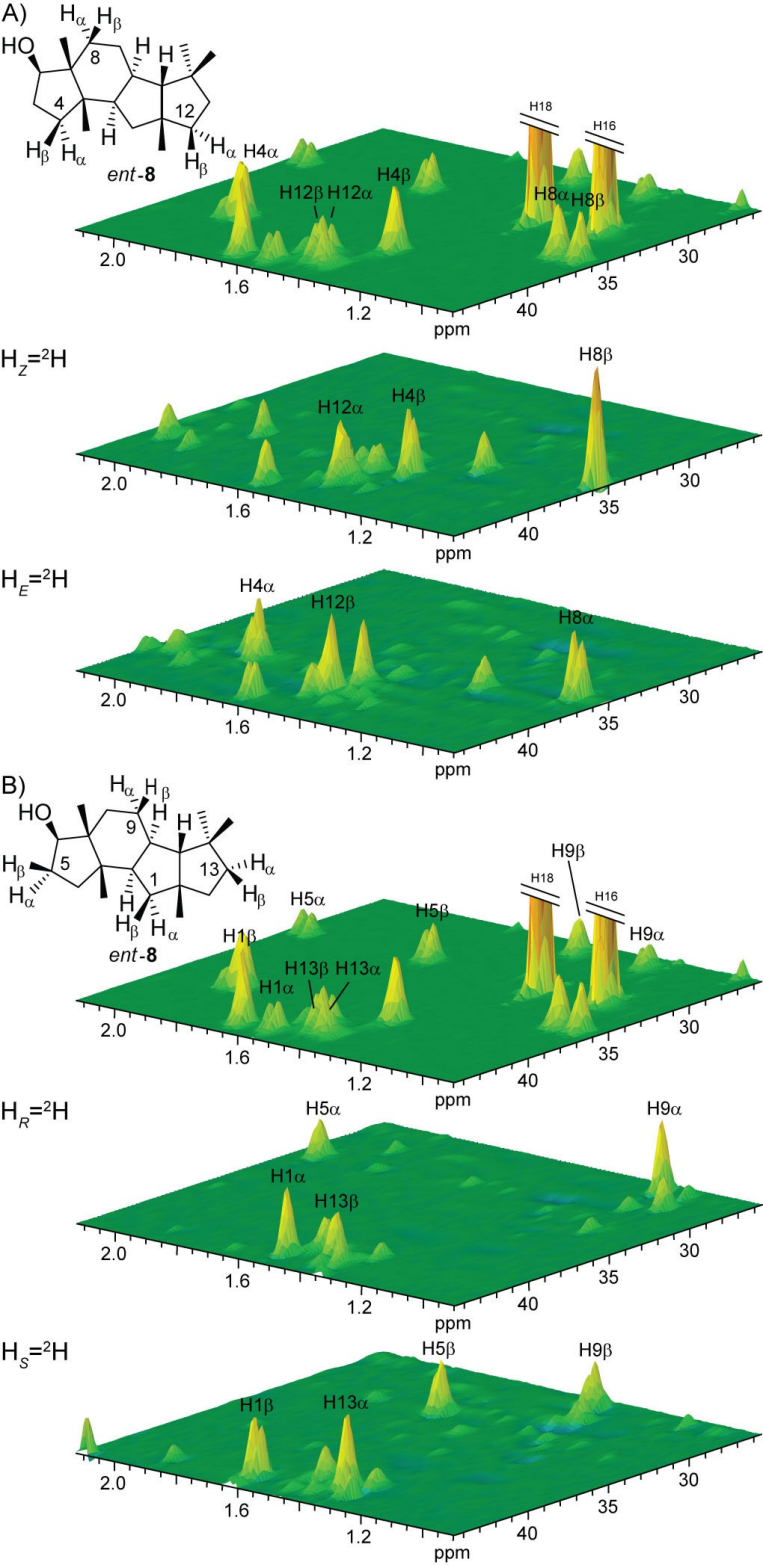
Determination of the absolute configuration of **8**. Partial HSQC spectra covering the methylene group area of unlabelled **8** (top) in comparison to extracts from incubation experiments with FgGS, FPPS, GGPPS and A) (*Z*)‐(4‐^13^C,4‐^2^H)IPP (middle) or (*E*)‐(4‐^13^C,4‐^2^H)IPP, and B) (*R*)‐(1‐^13^C,1‐^2^H)IPP (middle) or (*S*)‐(1‐^13^C,1‐^2^H)IPP with IDI showing selective incorporation of deuterium atoms, as expected for *ent*‐**8** (cf. Scheme 2 B and Figure S1).

In a second set of experiments, the substrates (*R*)‐ and (*S*)‐(1‐^13^C,1‐^2^H)IPP^18^ were used in conjunction with the isopentenyl diphosphate isomerase (IDI) from *Escherichia coli*,^18^, ^19^ FPPS and GGPPS. The resulting GGPP isotopomers labelled at C1, C5, C9 and C13 were converted by FgGS to give enantioselectively deuterated products whose HSQC spectra pointed to the same absolute configuration of *ent*‐**8** (Figure 1 B). Conclusively, the absolute configuration of fusaterpenol needs revision to (2*R*,3*S*,6*R*,7*R*,10*R*,11*R*,14*R*)‐**8**.

The proposed biosynthesis of **8** from GGPP through cyclisation to cation **A**, skeletal rearrangement to **B**, 2,10‐cyclisation to **C**, 3,7‐cyclisation and likely simultaneous attack of water to avoid a secondary cation intermediate was further studied by the enzymatic conversion of all 20 isotopomers of (^13^C)GGPP^11^ (Table S2) with FgGS;^8^ the ^13^C label was incorporated into the expected positions in all cases (Figure 2). In particular, a clear stereochemical course for the geminal methyl groups C16 and C17 of GGPP was observed, with C16 ending up in the *pro*‐*S* and C17 in the *pro*‐*R* position, thus suggesting that the substrate is bound to the enzyme's active site with a precisely defined conformational fold. The same stereochemical course has previously been reported for the bacterial diterpenes **1**, **13**, **14** and **15** based on the same ^13^C‐labelling experiments, whereas **8** is the first fungal compound for which this has been studied (cf. labels for C16 and C17 in Scheme 1). For the fungal sesterterpenes **6** and **7**, a related stereochemical course is obvious because the additional prenyl unit of GFPP compared to GGPP is attached to the *E* position, equal to C16.

**Figure 2:**
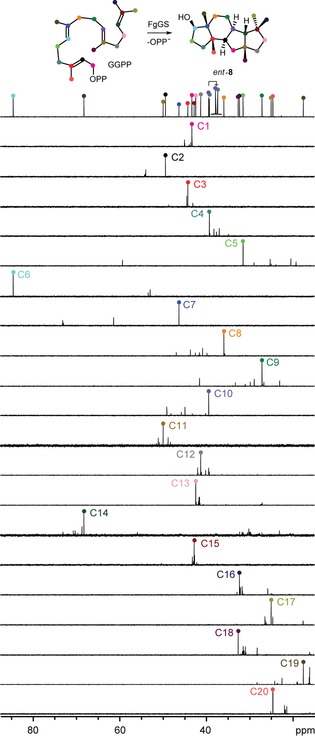
Investigation of the cyclisation mechanism towards *ent*‐**8** by ^13^C_1_ labelling. Partial ^13^C NMR spectra of unlabelled *ent*‐**8** (top) compared to conversions of all ^13^C_1_ isotopomers of GGPP by FgGS. Matching signals originating from the same carbon atoms are coloured the same way.

To further investigate this point for fungal enzymes, the gene of a bifunctional TS+PT enzyme from *A. brasiliensis* CBS 101.740 (accession no. OJJ72250) with the published variediene synthase EvVS^5^ as closest characterised homologue (Figure 3, 65 % identical residues) was cloned and expressed in *E. coli* (Table S3, Figures S9 and S10). The purified enzyme converted a combination of DMAPP and IPP, and also GGPP, into **3** as a single product whose structure was resolved by NMR spectroscopy (Table S4 and Figures S11–S18), thus identifying this enzyme as *A. brasiliensis* variediene synthase (AbVS). This finding suggests that the whole clade of enzymes shaded in grey in Figure 3 might have activity as variediene synthases. The absolute configuration was investigated by using the enantioselective‐deuteration approach and independently by optical rotary power measurement; both pointed to the same configuration as published for **3** from *E. variecolor* (Figures S19 and S20).^5^ The stereochemical fate of the geminal methyl groups C16 and C17 was in agreement with the observations made for the previously investigated diterpene synthases (Scheme 1, Figure S21). In summary, all these data point to a very similar conformation of GGPP in the enzymes’ active sites that explains a common reaction via **A** and **B** for all the observed products.

**Figure 3:**
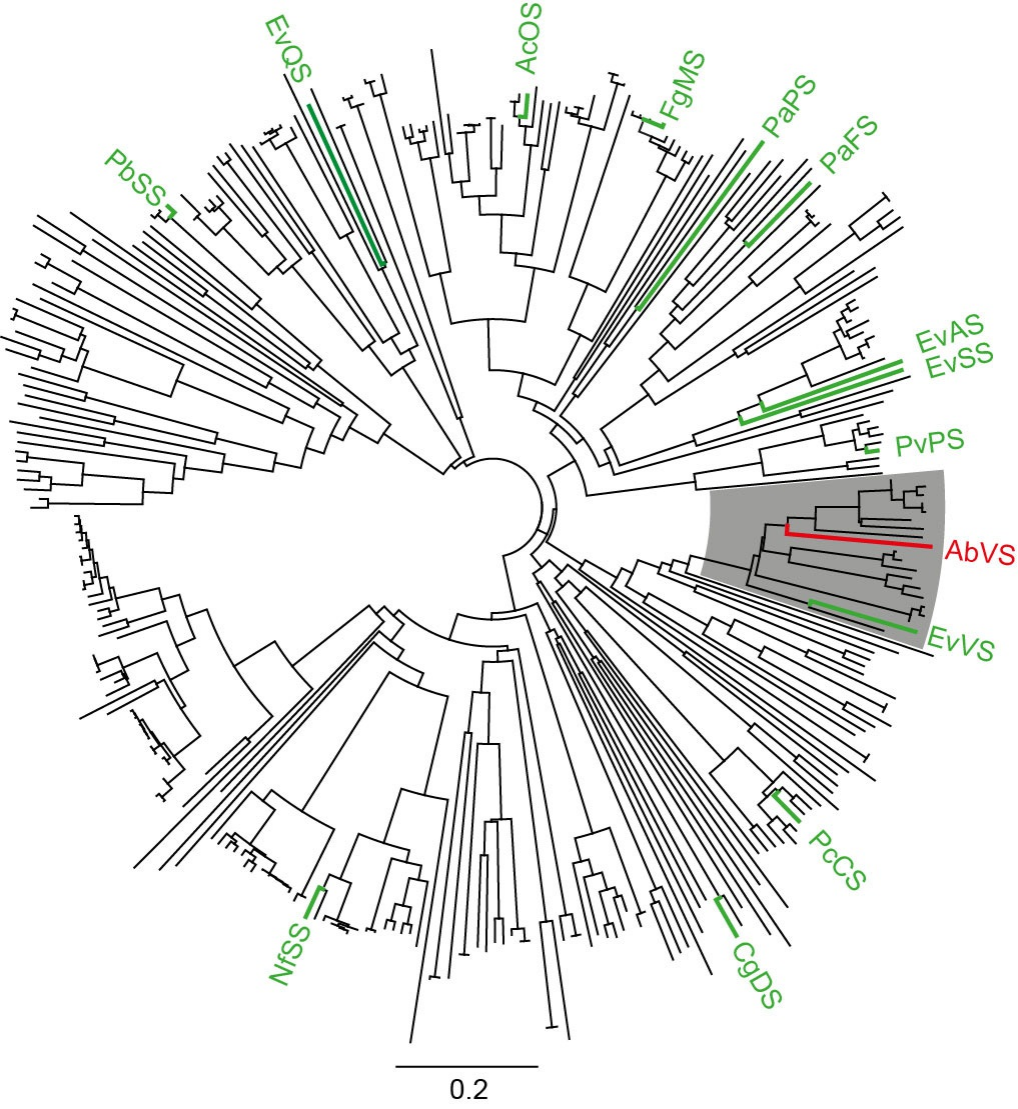
Phylogenetic tree constructed from 319 fungal bifunctional TS+PT enzymes. Previously characterised enzymes are shown in green, the enzyme characterised in this study is shown in red. AcOS: *Aspergillus clavatus* ophiobolin F synthase,^20^ CgDS: *Colletotrichum gloeosporioides* dolasta‐1(15),8‐diene synthase,^21^ EvAS: *E. variecolor* astellifadiene synthase,^22^ EvQS: *E. variecolor* quiannulatene synthase,^23^ EvSS: *E. variecolor* stellata‐2,6,19‐triene synthase,^24^ EvVS: *E. variecolor* variediene synthase,^5^ FgMS: *F. graminearum* mangicdiene synthase,^8^ NfSS: *Neosartorya fischeri* sesterfisherol synthase,^25^ PaFS: *P. amygdali* fusicocca‐2,10(14)‐diene synthase,^26^ PaPS: *P. amygdali* phomopsene synthase,^4^ PbSS: *Penicillium brasilianum* sesterbrasiliatriene synthase,^27^ PcCS: *P. chrysogenum* cyclopiane‐type diterpene synthase,^6^ PvPS: *Penicillium verruculosum* preasperterpenoid A synthase.^27^

During the GC/MS analysis of purified **3**, the formation of small amounts of a new compound by a thermal reaction in the injector was observed (Figure S22). This was also indicated by a minor, but clearly observable fronting for the peak of **3**, probably caused by the same thermal reaction at elevated elution temperatures. The mass spectrum was very similar to the mass spectrum of **3**, thus suggesting a closely related structure for the thermal reaction product, such as **16**, that could arise by Cope rearrangement of **3** (Scheme 3). A similar gas chromatographic behaviour can be observed for germacrene A, which undergoes Cope rearrangement to β‐elemene, with both compounds exhibiting highly similar EI mass spectra.^18^, ^28^, ^29^, ^30^ To identify the product of the thermal reaction, a solution of purified **3** in nitrobenzene was heated to 210 °C, resulting in the formation of the same product as observed in the GC/MS analysis. The compound was isolated, and its structure was elucidated by NMR spectroscopy as the 6*Z* isomer of variediene (**17**, Table S5 and Figures S23–S30) instead of expected **16**. To clearly distinguish between the known variediene isomers **3**, **9** and **17**, we suggest the names (2*Z*,6*E*)‐varie‐2,6‐diene (**3**, “variediene”), (6*E*)‐varie‐2(15),6‐diene (**9**, termed GJ1012C in the original publication)^8^ and (2*Z*,6*Z*)‐varie‐2,6‐diene (**17**).

**Scheme 3:**
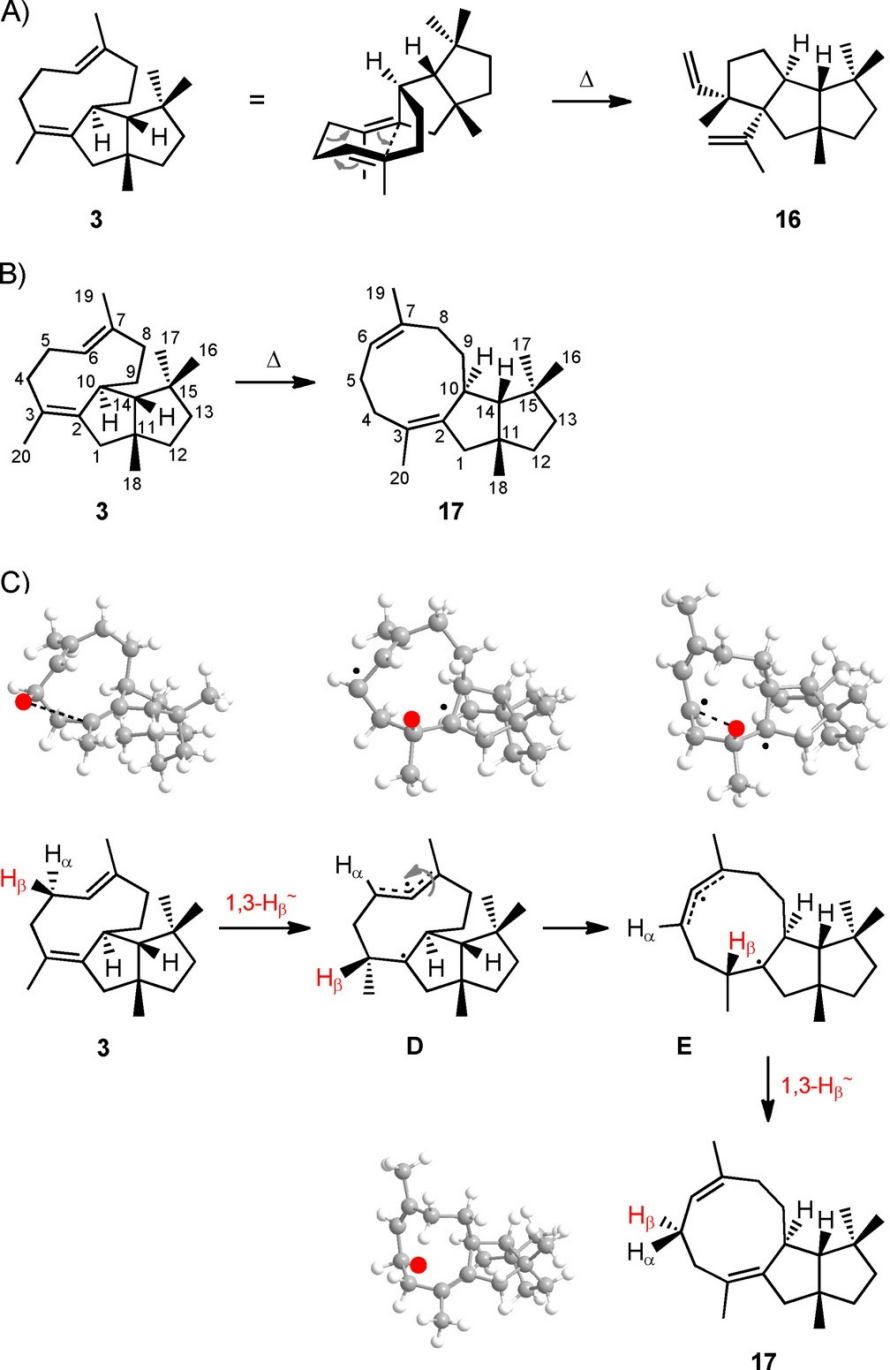
Thermal reactions of **3**. A) Hypothetical Cope rearrangement to **16**, B) experimentally verified *E*/*Z* isomerisation to **17** and C) hypothetical mechanism for the involvement of the C5 hydrogen atoms during the isomerisation process leading to a partial epimerisation. Models generated by Chem3D Ultra 13.0 (MM2).

To strengthen the stereochemical assignment of the methylene groups in **17**, especially of those that are part of the nine‐membered ring, enantioselectively deuterated samples of **3** were converted to **17** and analysed by HSQC (Figures S31 and S32). Interestingly, the stereogenic centre at C5 showed partial epimerisation, whereas the stereocentres at all other deuterated carbons were configurationally stable during the thermal isomerisation (only for C9 did a signal overlap prevent conclusions). Therefore, the hydrogens bound to C5 apparently take part in the isomerisation mechanism. Although the majority of molecules of compound **3** might react in a simple *E*/*Z* isomerisation by distortion of the C6=C7 olefin with retention of configuration at C5, a possible explanation for the partial epimerisation at this carbon involves a hydrogen transfer of H5_β_ to C3, resulting in biradical **D** with a C5–6–7‐centred allyl radical and a radical at C2 (Scheme 3 C). The allyl radical can then undergo *E*/*Z* isomerisation to **E**, followed by a back transfer of H5_β_ that might proceed with attack at C5 from the opposite face, thus explaining the overall partial epimerisation at this carbon.

In summary, we have revised the absolute configuration of fusaterpenol (**8**), such that it is now in line with the absolute configurations of related diterpenes. A newly characterised variediene synthase from *A. brasiliensis* was also found to produce variediene with matching absolute configuration. Together with the stereochemical fates of the geminal methyl groups C16 and C17, which is the same for all previously investigated diterpene synthases, and the variediene synthase described here, a common conformational fold of GGPP in the active sites of all diterpene synthases making the products shown in Scheme 1 can be assumed. Furthermore, an interesting thermal *E*/*Z* isomerisation of variediene was found. Only by stereoselective deuterium labelling did a surprising mechanism that involves epimerisation at the neighbouring carbon become evident; this impressively demonstrates the high utility of stereoselective isotope labelling beyond biosynthetic and enzyme mechanistic investigations.

## Conflict of interest

*The authors declare no conflict of interest*.

## Supporting information

As a service to our authors and readers, this journal provides supporting information supplied by the authors. Such materials are peer reviewed and may be re‐organized for online delivery, but are not copy‐edited or typeset. Technical support issues arising from supporting information (other than missing files) should be addressed to the authors.

Supplementary
