# Supplementary material for: A Family of Related Fungal and Bacterial Di‐ and Sesterterpenes: Studies on Fusaterpenol and Variediene
Source: Chembiochem. 2019 Nov 7;21(4):486–91. doi: 10.1002/cbic.201900462 (PMC7065159; doi:10.1002/cbic.201900462)
Supplement: Supplementary file 1 — Supplementary [file CBIC-21-486-s001.pdf]

CHEMBIOCHEM

Supporting Information

A Family of Related Fungal and Bacterial Di- and Sesterterpenes: Studies on Fusaterpenol and Variediene

Jan Rinkel,[a] Simon T. Steiner,[a] Guangkai Bian,[b] Rong Chen,[b] Tiangang Liu,[b] and Jeroen S. Dickschat*[a]

cbic_201900462_sm_miscellaneous_information.pdf

Gene expression and protein purification
Expression and purification of FgGS was performed according to the literature-known procedure[1] with few modifications. A preculture of *E. coli* BL21(DE3) transformed with pET28a-FgGS[1] was grown in LB medium with kanamycin (50 µg mL^{-1}) overnight while shaking at 37 °C. A gene expression culture inoculated with the preculture (1/1000) was grown in LB medium with kanamycin (50 µg mL^{-1}) shaking at 37 °C until OD_{600} = 0.4-0.6 was reached. After cooling to 18 °C enzyme expression was induced by addition of aqueous IPTG solution (400 mM, 1/1000). The culture was shaken at 18 °C overnight and cells were harvested via centrifugation (9800 g, 5 min, 4 °C). The cells were lysed by ultra-sonication (3x 1 min, 50% power) on ice, after resuspending the cell pellet with binding buffer (10 mL L^{-1}; 20 mM Na_2HPO_4, 0.5 M NaCl, 20 mM imidazole, 1 mM $MgCl_2$, pH 7.4). The cell debris were removed by centrifugation (14600 g, 7 min, 4 °C) and the soluble protein fractions were loaded to Ni^{2+}-NTA superflow affinity chromatography columns by Qiagen (Venlo, Netherlands) equilibrated with binding buffer. The columns were washed with binding buffer (2x 10 mL L^{-1} culture) and the desired proteins were eluted with elution buffer (2x 10 mL L^{-1} culture; 20 mM Na_2HPO_4, 0.5 M NaCl, 500 mM imidazole, 1 mM $MgCl_2$, pH 7.4). The obtained fraction was directly used for incubation experiments.

Enzyme reaction and product isolation
For preparative isolation of *ent*-**8**, a large scale incubation was done by dissolving GGPP (trisammonium salt, 100 mg) in substrate buffer (20 mL; 25 mM NH_4HCO_3). This solution was slowly added within 2 h to a stirred mixture of protein preparations of recombinant FgGS (100 mL; from 8 L expression culture), binding buffer (80 mL) and incubation buffer (200 mL; 50 mM Tris/HCl, 10 mM $MgCl_2$, 20% glycerol, pH = 8.2).
The reaction mixture was incubated overnight at 28 °C and was extracted with pentane (2x 200 mL), the extracts were dried with $MgSO_4$ and concentrated in vacuo. Column chromatography on silica gel [pentane/Et_2O (4:1)] yielded diterpene *ent*-**8** (1.78 mg) as a white solid. GC/MS and NMR data recorded in C_6D_6 (Table S1 and Figures S1–S8) matched the previous assignment in $CDCl_3$[1] and identified the diterpene as fusaterpenol (GJ1012E, *ent*-**8**).

NMR spectroscopy
NMR spectra were recorded on a Bruker (Billerica, MA, USA) Avance I (300 MHz), Avance I (400 MHz), Avance I (500 MHz), Avance III HD Prodigy (500 MHz) or an Avance III HD Cryo (700 MHz) NMR spectrometer. Spectra were referenced against solvent signals (^{1}H-NMR, residual proton signals: C_6D_6 δ = 7.16; ^{13}C-NMR: C_6D_6 δ = 128.06.[2]

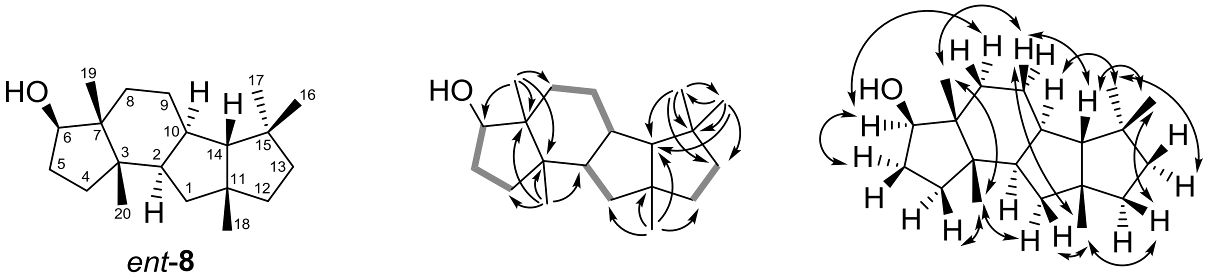

Figure S1. Structure elucidation of fusaterpenol (*ent*-**8**) including methylene group assignment. H,H-COSY correlations are shown in bold grey, single headed arrows represent HMBC correlations and NOESY correlations are shown by double headed arrows.

Table S1. NMR spectral data of fusaterpenol (*ent*-**8**) in C_6D_6 recorded at 298 K.

C[a]	^{13}C[b]		^{1}H[b]
1	43.4	CH_2	1.65 (m, H_β)
			1.56 (m, H_α)
2	49.5	CH	1.90 (m)
3	44.3	C_q	–
4	39.3	CH_2	1.86 (m, H_α)
			1.37 (m, H_β)
5	31.6	CH_2	2.07 (m, H_α)
			1.67 (m, H_β)
6	84.6	CH	3.38 (ddd, ^3J = 7.7, ^3J = 2.7, J = 2.7, 1H)
7	46.4	C_q	–
8	36.0	CH_2	1.01 (ddd, ^2J = 13.5, ^3J = 13.5, J = 3.2, H_α)
			0.97 (m, H_β)
9	27.2	CH_2	1.42 (m, H_β)
			1.20 (m, H_α)
10	39.5	CH	1.90 (m)
11	50.0	C_q	–
12	41.3	CH_2	1.53 (m, H_β)
			1.49 (m, H_α)
13	42.5	CH_2	1.45 (m, H_β)
			1.41 (m, H_α)
14	68.3	CH	1.27 (s, 1H)
15	42.8	C_q	–
16	32.4	CH_3	1.06 (s, 3H)
17	25.1	CH_3	0.95 (s, 3H)
18	32.7	CH_3	1.25 (s, 3H)
19	17.6	CH_3	1.00 (s, 3H)
20	24.7	CH_3	1.15 (s, 3H)

[a] Carbon numbering as shown in Figure S1. [b] Chemical shifts δ in ppm, multiplicity: s = singlet, d = doublet, m = multiplet, coupling constants J are given in Hertz.

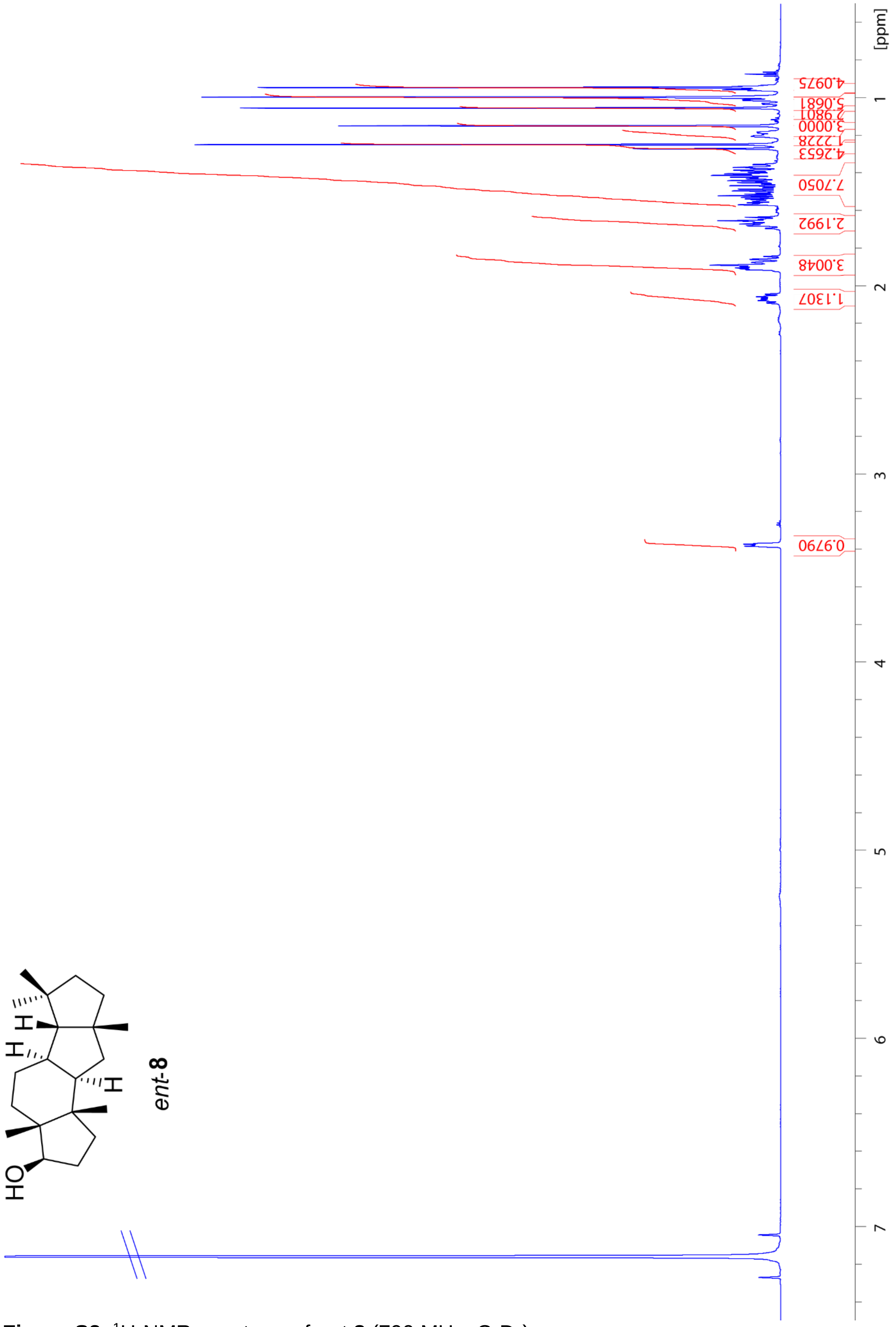

Figure S2. ^{1}H-NMR spectrum of *ent*-**8** (700 MHz, C_6D_6).

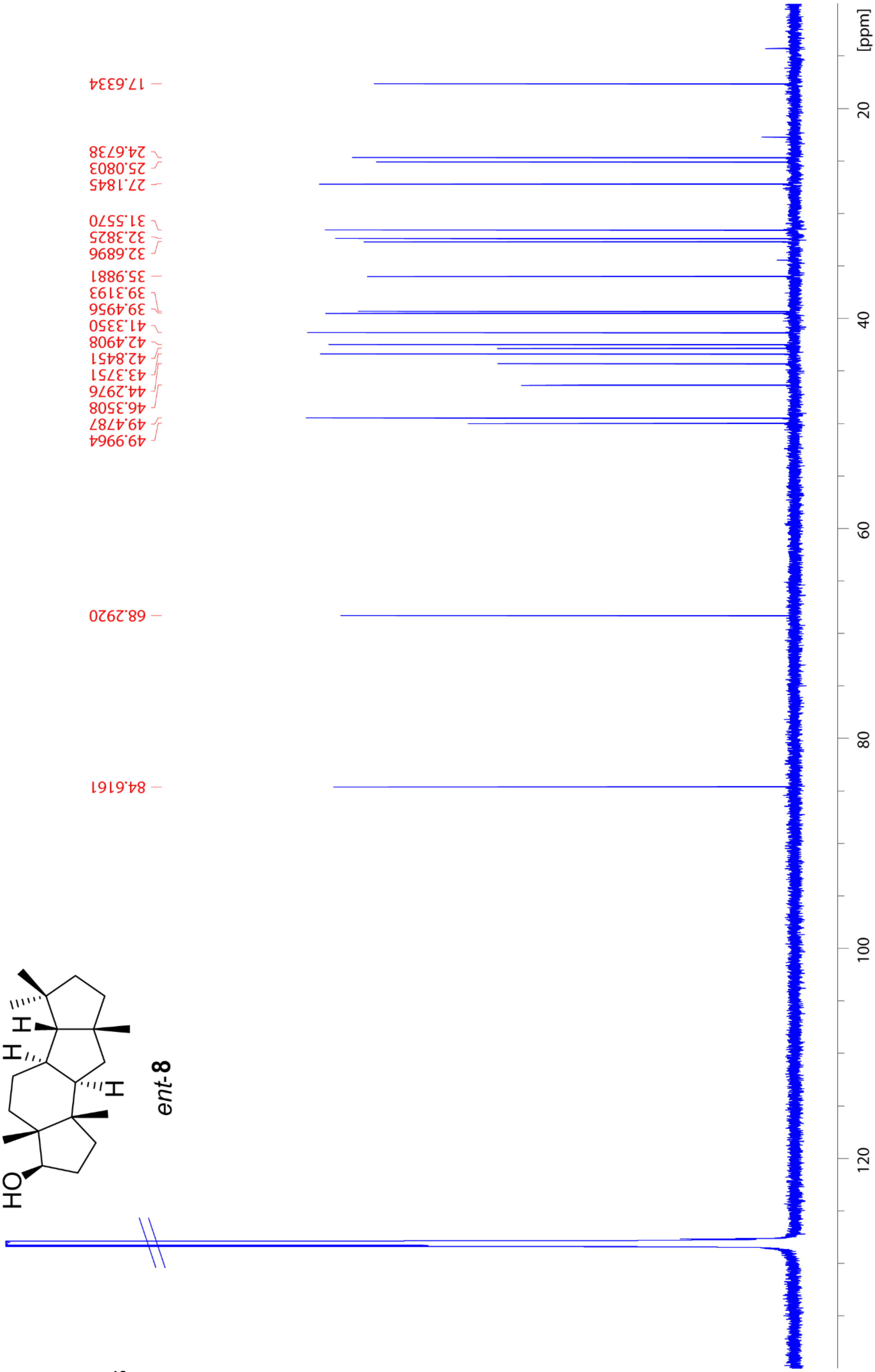

Figure S3. ^{13}C-NMR spectrum of *ent*-**8** (175 MHz, C_6D_6).

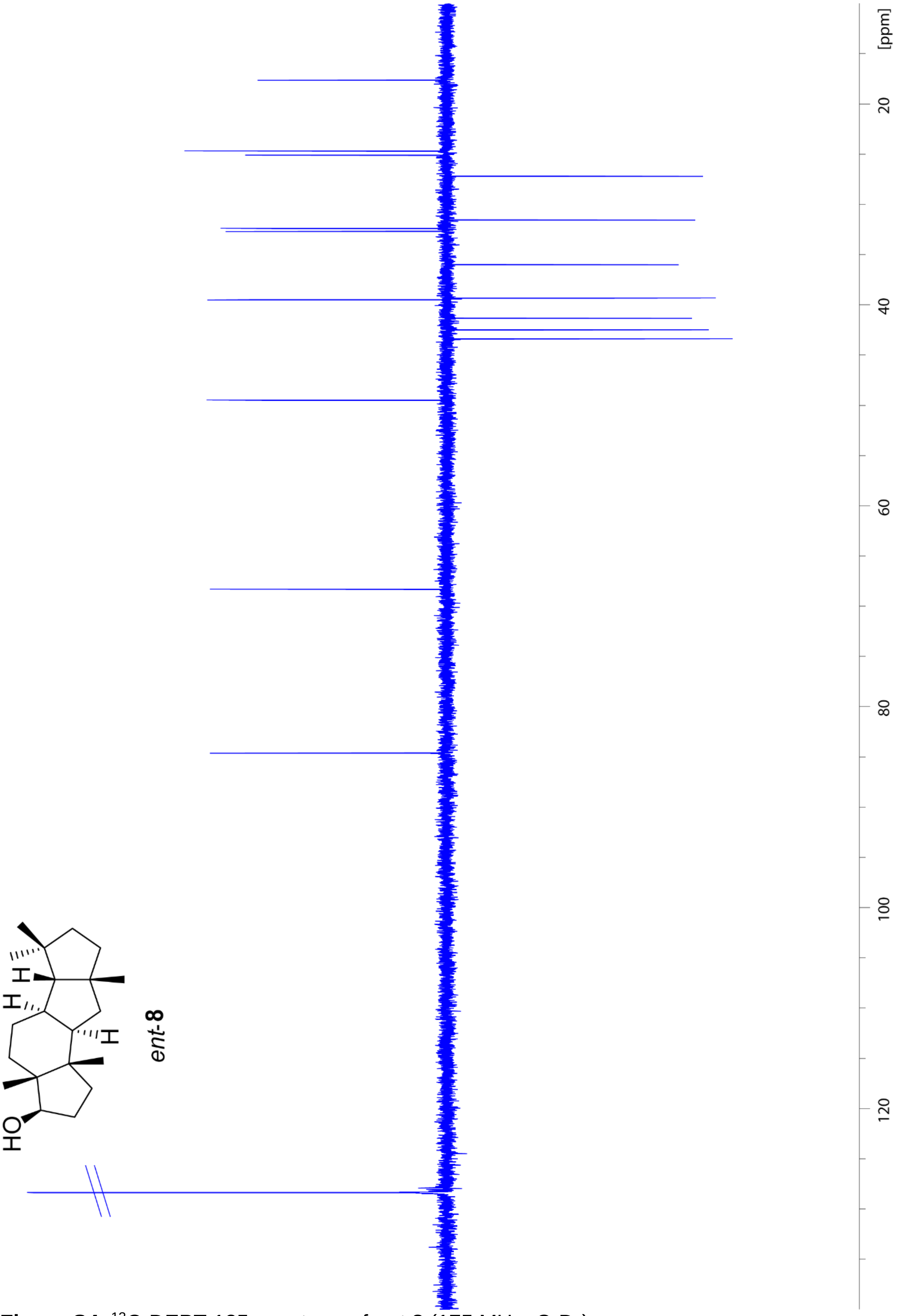

Figure S4. ^{13}C-DEPT 135 spectrum of *ent*-**8** (175 MHz, C_6D_6).

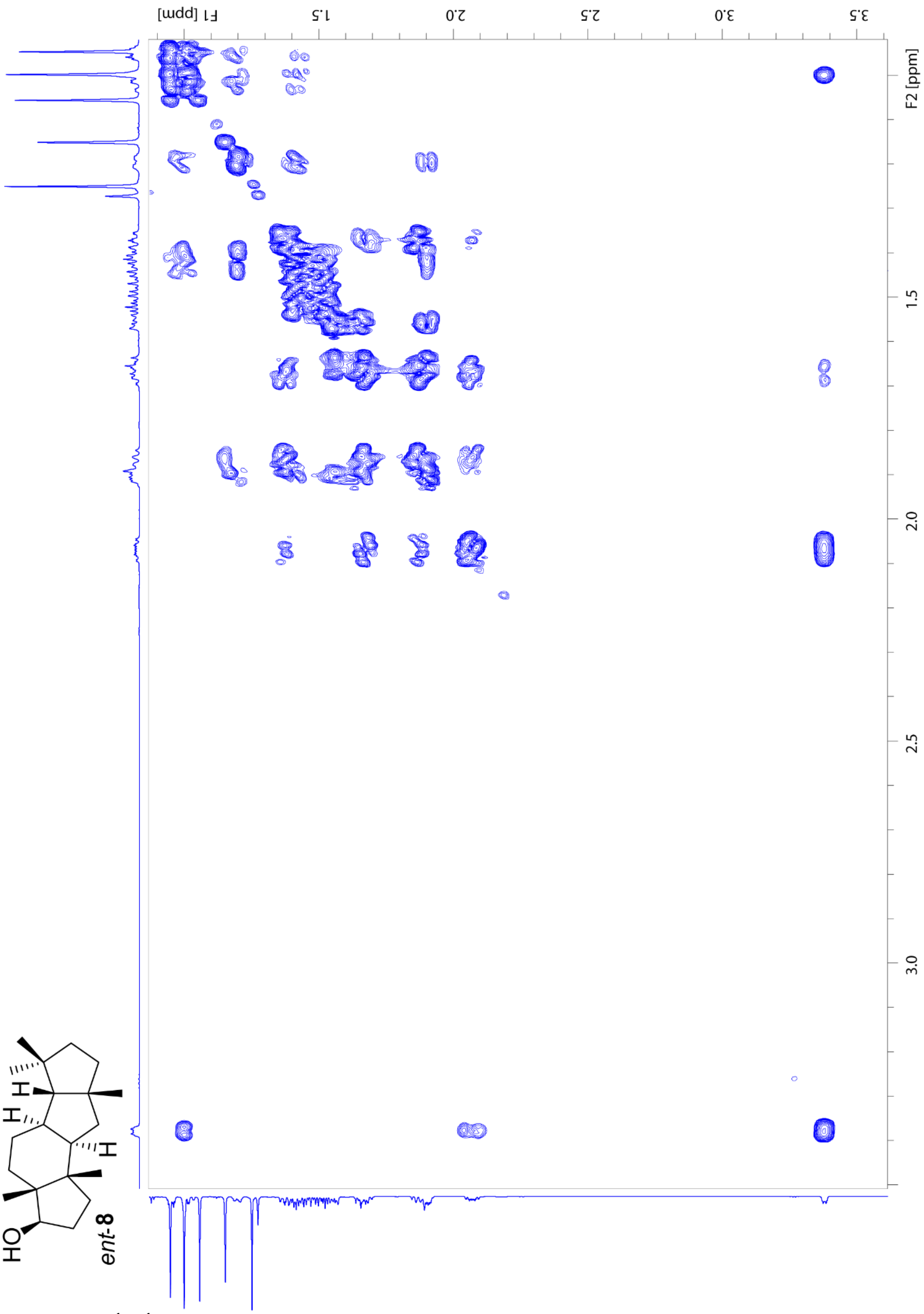

Figure S5. ^{1}H,^{1}H-COSY spectrum of *ent*-**8** (700 MHz, C_6D_6).

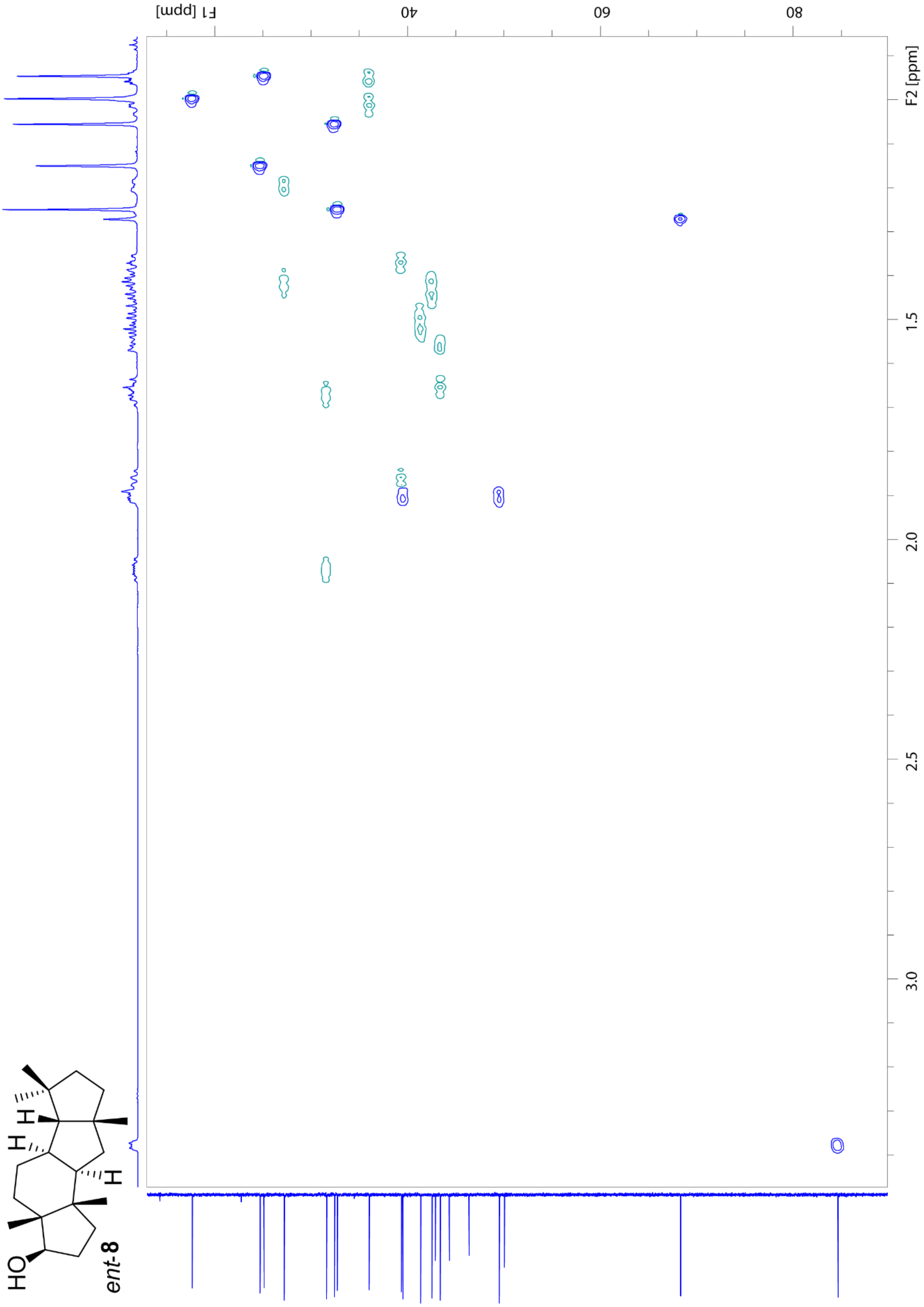

Figure S6. HSQC spectrum of *ent*-**8** (700 MHz, C_6D_6).

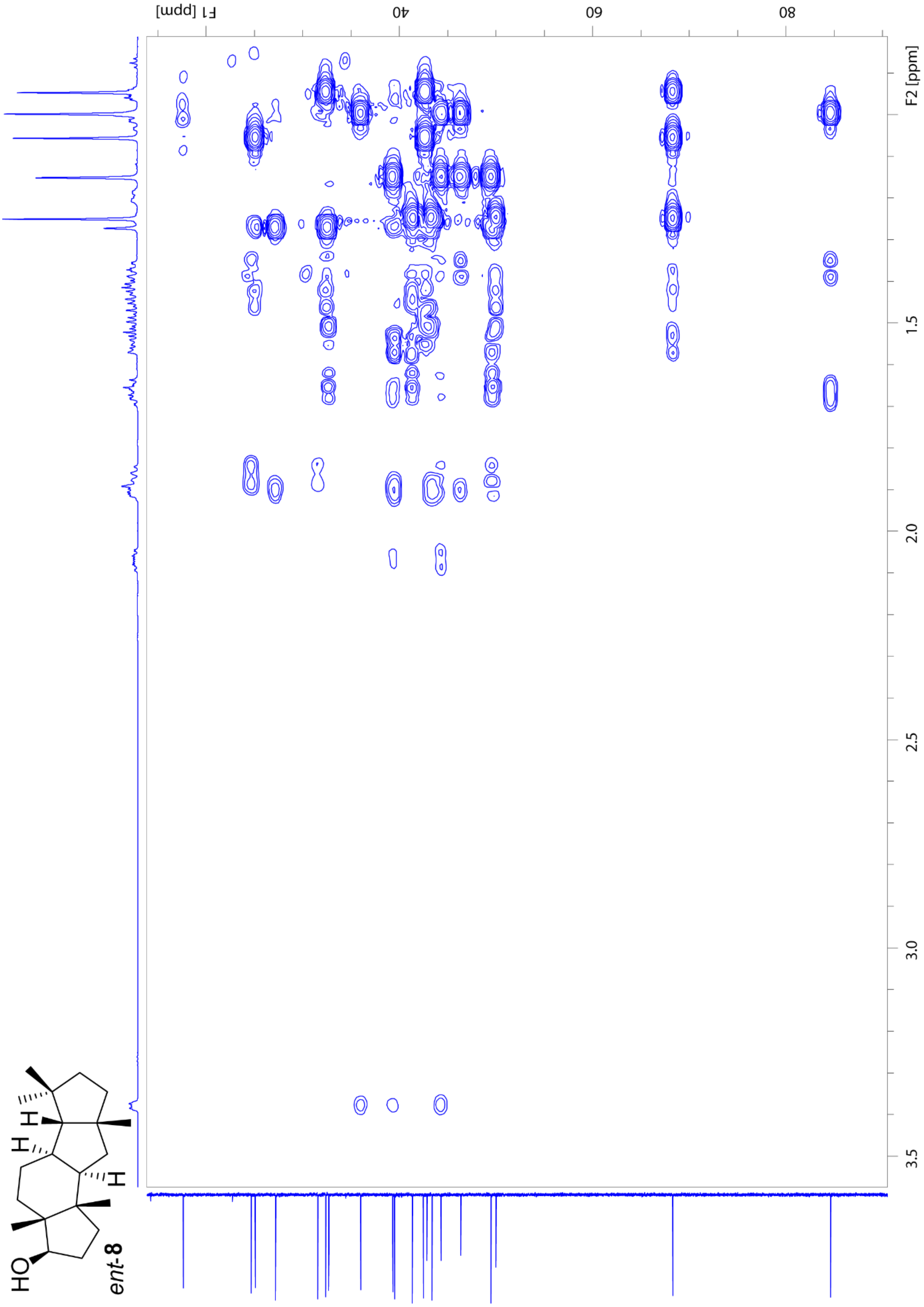

Figure S7. HMBC spectrum of *ent*-**8** (700 MHz, C_6D_6).

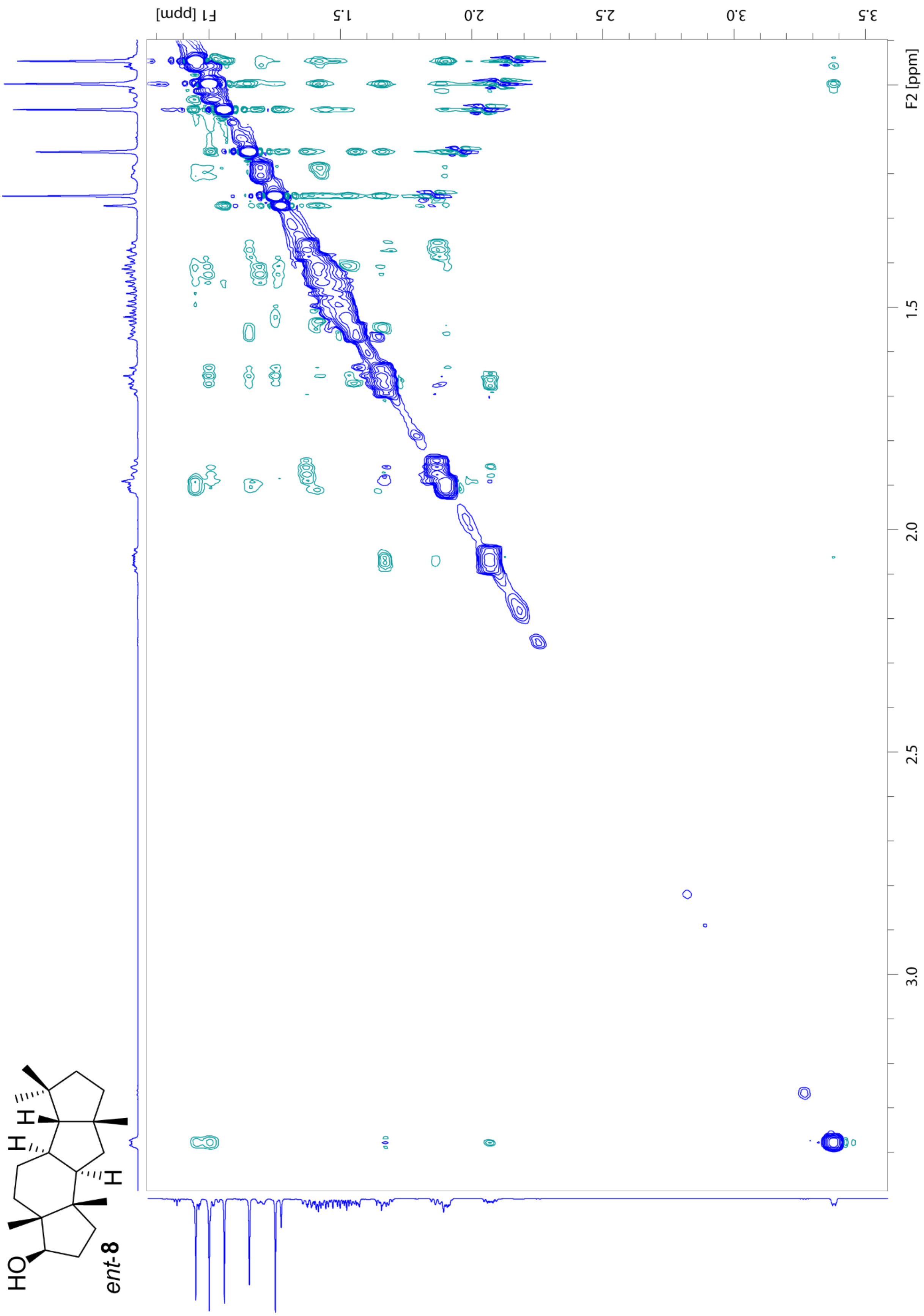

Figure S8. NOESY spectrum of *ent*-**8** (700 MHz, C_6D_6).

Incubation experiments with isotopically labelled substrates

Isotopic labelling experiments were performed with amounts of ca. 1 mg labelled diphosphate dissolved in substrate buffer (1 mL), incubation buffer (5 mL), enzyme elution fractions (1 mL of terpene synthase solution and 0.5 mL for each additional enzyme (FPPS, GGPPS, IDI) and binding buffer (to 10 mL total volume) with the substrates and enzyme preparations as listed in Table S2. After incubation with shaking at 28 °C for 3 h the products were extracted with C_6D_6 (650 μL and 300 μL) and analysed by NMR and GC/MS.

Table S2. Incubation experiments with isotopically labelled substrates.

carbons	substrate(s)	enzyme(s)	shown in
1	(1-^{13}C)IPP[3] + FPP	FgGS, GGPPS[3]	Figure 2
2	(2-^{13}C)IPP[4] + FPP	FgGS, GGPPS	Figure 2
3	(3-^{13}C)IPP[3] + FPP	FgGS, GGPPS	Figure 2
4	(4-^{13}C)IPP[3] + FPP	FgGS, GGPPS	Figure 2
5	(1-^{13}C)FPP[5] + IPP	FgGS, GGPPS	Figure 2
6	(2-^{13}C)FPP[5] + IPP	FgGS, GGPPS	Figure 2
7	(3-^{13}C)FPP[5] + IPP	FgGS, GGPPS	Figure 2
8	(4-^{13}C)FPP[5] + IPP	FgGS, GGPPS	Figure 2
9	(5-^{13}C)FPP[5] + IPP	FgGS, GGPPS	Figure 2
10	(6-^{13}C)FPP[5] + IPP	FgGS, GGPPS	Figure 2
11	(7-^{13}C)FPP[5] + IPP	FgGS, GGPPS	Figure 2
12	(8-^{13}C)FPP[5] + IPP	FgGS, GGPPS	Figure 2
13	(9-^{13}C)FPP[5] + IPP	FgGS, GGPPS	Figure 2
14	(10-^{13}C)FPP[5] + IPP	FgGS, GGPPS	Figure 2
15	(11-^{13}C)FPP[5] + IPP	FgGS, GGPPS	Figure 2
16	(12-^{13}C)FPP[5] + IPP	FgGS, GGPPS	Figure 2
17	(9-^{13}C)GPP[6] + IPP	FgGS, GGPPS	Figure 2
18	(10-^{13}C)GPP[7] + IPP	FgGS, GGPPS	Figure 2
19	(15-^{13}C)FPP[5] + IPP	FgGS, GGPPS	Figure 2
20	(20-^{13}C)GGPP[3]	FgGS, GGPPS	Figure 2
4, 8, 12	(*Z*)-(4-^{13}C,4-^{2}H)IPP[8] + DMAPP	FgGS, GGPPS, FPPS[9]	Figure 1A
4, 8, 12	(*E*)-(4-^{13}C,4-^{2}H)IPP[8] + DMAPP	FgGS, GGPPS, FPPS	Figure 1A
1, 5, 9, 13	(*R*)-(1-^{13}C,1-^{2}H)IPP[10]	FgGS, GGPPS, FPPS, IDI[10]	Figure 1B
1, 5, 9, 13	(*S*)-(1-^{13}C,1-^{2}H)IPP[10]	FgGS, GGPPS, FPPS, IDI	Figure 1B
4, 8, 12	(*Z*)-(4-^{13}C,4-^{2}H)IPP + DMAPP	AbVS, GGPPS, FPPS	Figure S19
4, 8, 12	(*E*)-(4-^{13}C,4-^{2}H)IPP + DMAPP	AbVS, GGPPS, FPPS	Figure S19
1, 5, 9, 13	(*R*)-(1-^{13}C,1-^{2}H)IPP	AbVS, GGPPS, FPPS, IDI	Figure S20
1, 5, 9, 13	(*S*)-(1-^{13}C,1-^{2}H)IPP	AbVS, GGPPS, FPPS, IDI	Figure S20
16	(12-^{13}C)FPP + IPP	AbVS, GGPPS	Figure S21
17	(9-^{13}C)GPP + IPP	AbVS, GGPPS	Figure S21

Strains and culture conditions

Aspergillus brasiliensis CBS 101.740 was obtained from the CBS culture collection (Westerdijk Fungal Biodiversity Institute, Utrecht, The Netherlands) and cultured in CZA liquid medium (30.0 g saccharose, 2.00 g $NaNO_3$, 1.00 g K_2HPO_4, 0.50 g $MgSO_4$, 0.5 g KCl, 0.01 g $FeSO_4$, 1 L demineralised water, pH 7.2) for 7d at 22°C under exclusion of daylight for the isolation of mRNA.

Gene cloning

mRNA was isolated using RNeasy Plant Mini Kit by Qiagen (Venlo, Netherlands). To remove additional DNA, DNase I (RNase-free) of New England BioLabs Inc. (Ipswich, USA) was applied. Purified RNA was transcribed to cDNA using SuperScript™ III Reverse Transcriptase by invitrogen (Carlsbad, USA). These steps were performed following the experimental procedures provided by the companies. cDNA was stored at 4 °C and used as a template for gene amplification. The target gene (accession number OJJ72250) was amplified by PCR using Q5® High-Fidelity DNA Polymerase by New England BioLabs Inc. (Ipswich, USA) and primers LB044f and LB044r (Table S1). PCR conditions were: Initial denaturation at 98 °C for 5 min; 33 cycles with melting at 98 °C for 10 s, annealing at 68 °C for 45 s and elongation at 72 °C for 30 s; final elongation at 72 °C for 5 min. The obtained PCR product was elongated with pYE-Express homology arms in a second PCR using primers ST021f and ST021r under the same conditions. The elongated PCR product was used for homologous recombination in yeast[11] with pYE-Express shuttle vector[12] which was linearized by digestion with HindIII and EcoRI. The transformed yeast cells were cultured for 3 days at 28 °C on SM-URA agar plates (425 mg yeast nitrogen base, 1.25 g ammonium sulphate, 5 g glucose, 192.5 mg nutritional supplement minus uracil, 5 g agar, 250 mL water), harvested and plasmid DNA was extracted using the Zymoprep Yeast Plasmid Miniprep II kit (Zymo Research, Irvine, CA, USA). The plasmid DNA was used for electroporation of E. coli BL21(DE3) electrocompetent cells which were spread on LB agar plates (10.0 g tryptone, 5.0 g yeast extract, 5.0 g NaCl, 16.0 g agar, 1 L water, pH 7.2) and cultured at 37 °C overnight. A single colony was selected and used to inoculate LB-medium containing kanamycin (50 µg mL^{-1}). The colonies were grown for 24 h. Plasmid DNA was isolated using PureYield® Plasmid Miniprep System by Promega Corp. (Madison, USA). The correct incorporation of the target genes was verified by analytic digest with PvuII and XhoI, as well as gene sequencing to yield plasmid pYE-OJJ72250 (AbVS).

Table S3. Primers used for gene cloning.

Primer	Sequence
LB044f	ATGGTCCCTACAAGTCTATCC
LB044r	TCAGACTTCCAATCGGTGAAC
ST021f	GGCAGCCATATGGCTAGCATGACTGGTGGAATGGTCCCTACAAGTCTATCC
ST021r	TCTCAGTGGTGGTGGTGGTGGTGCTCGAGTTCAGACTTCCAATCGGTGAAC

MVPTSLSPDDTSDPVPRSSSDIQGFCHNYPLRRHKYEDQANKGSQQCRDDWEQYIGPIERWGCGNPWE
GHFAAVVLPFCRPDRIAIISYCFEYAFMYDNVVESAAKSTVNINRDDIALDETEYRTVRSVTGTKQIQ
SKMLLDLLSIDPVCAEVVIDSWKTMIDTTVKQDKTRTFSNLEEYVDFRIIDTGAPFVDTLMRFGMNIL
LTPEEEELVAPIVKPCYAALGLANDYFSFDIEWEEFQQPESNQSTMTNAVWLFMQWHQVDEQEAKRRV
RQVTNDYEREYQQRVRDFISGEGKSNTKLQLYLTALGYQIPGNIAWSLRCPRYHPWLCEEGSALLRAS
MDEARDVCNEGKRRSISGDSISSESSVWSGASDRSARSSVSSAPSLDEGKEPDRVMLGTEHLLGPAEY
IASLPSKGVREAFIDALNVWLVLPDRFVGVIKSIAKTLHNASLMLDDIEDGSPLRRGQPATHTIFGQA
LTINSANFVLIQAMDQVRQLEDSRCLDIFVEEMRNLFIGQSFDLYWTRQDECPSEEEYREMIRQKTGG
LFRLVARLMMQKATLKKNQHISLEPLVDLMGEYFQIRDDYKNLTEEYTGQKGFCEDLDEGKFSFPLIH
AHKLLPEWSEIRLLLQQGRQSGGLDVTQKQLVLGRLHDSGSMAYTEKTLRGLMGEIRLRIDQVEKESG
CSNWVLKLLVHRLEV

Figure S9. Amino acid sequence of AbVS (OOJ72250).

Gene expression and protein purification

Expression of AbVS from an *E. coli* BL21 (DE3) culture carrying the plasmid pYE-OJJ72250 was performed according to the same procedure as described above for FgGS. The obtained fraction was checked by SDS-PAGE (Figure S10) and directly used for incubation experiments. Protein concentrations were determined by Bradford assay (calibrated with bovine serum albumin).[13] Typical concentrations were 1.48 mg mL^{-1} for AbVS.

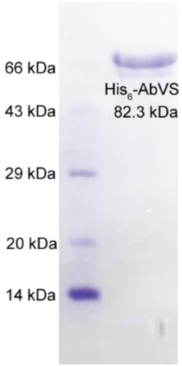

Figure S10. SDS-PAGE analysis of recombinant *Aspergillus brasiliensis* Variediene Synthase (His_6-AbVS, calculated molecular weight: 82.3 kDa).

APCI-MS measurements

High resolution mass spectra using APCI were recorded on an Orbitrap XL instrument (Thermo Fisher Scientific, Waltham, MA, USA).

Identification of AbVS as variediene synthase and product isolation

Test incubations to identify the substrate scope of recombinant AbVS were performed with GPP, FPP, GGPP, GFPP or DMAPP and IPP (1 mg) dissolved in substrate buffer (1 mL) and diluted with binding buffer (2.5 mL) and incubation buffer (4 mL). Protein preparations (0.5 mL) obtained from 100 mL expression culture were added, followed by incubation with shaking at 28 °C for 4 h. The products were extracted with hexane (100 µL), the extracts were dried with $MgSO_4$ and analysed by GC/MS. Only the incubations with GGPP and DMAPP with IPP showed the production of variediene (**3**).

For preparative isolation of **3**, large scale incubations were done by dissolving GGPP (trisammonium salt, 80 mg) in substrate buffer (20 mL). This solution was diluted by binding buffer (80 mL) and incubation buffer (200 mL). To start the conversion, AbVS elution fraction (100 mL; from 8 L expression culture) was added and the reaction mixtures were incubated for 3 h at 28 °C and were extracted with pentane (2x 200 mL), the extracts were dried with $MgSO_4$ and concentrated in vacuo. Column chromatography on silica gel with pentane yielded the pure diterpene (1.82 mg) as a colorless oil. GC/MS and NMR data measured in C_6D_6 (Table S3 and Figures S11–S18) were comparable to published data in $CDCl_3$[14] and identified the diterpene as variediene (**3**). The absolute configuration of the AbVS product was determined by optical rotary power measurement ($[\alpha]_D^{23}$ = –58.3 (C_6H_6, *c* 0.18), lit. $[\alpha]_D^{23}$ = –50.2 (C_6H_6, *c* 1)[14] and independently by isotopic labelling experiments (Figures S19–S20).

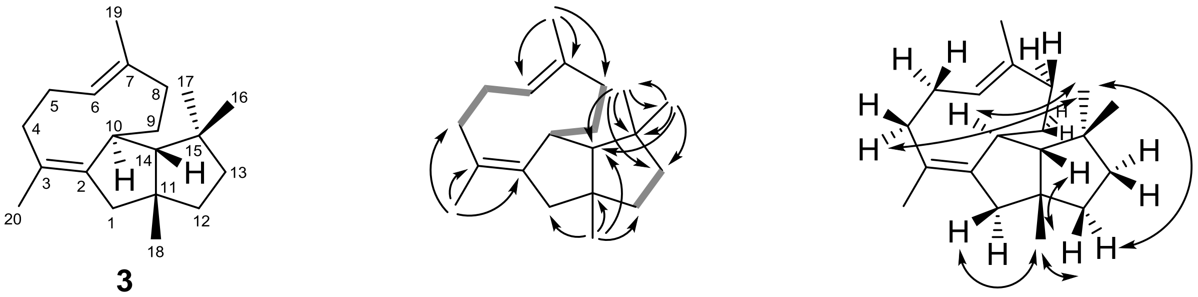

Figure S11. Structure elucidation of variediene (**3**) including methylene group assignment. H,H-COSY correlations are shown in bold grey, single headed arrows represent HMBC correlations and NOESY correlations are shown by double headed arrows.

Table S4. NMR spectral data of variediene (**3**) in C_6D_6 recorded at 298 K.

C[a]	^{13}C[b]		^{1}H[b]	^{13}C[c]
1	45.1	CH_2	2.18 (m, H_α)<br>2.11 (m, H_β)	44.99
2	144.2	C_q	–	144.08
3	121.2	C_q	–	121.01
4	38.3	CH_2	1.94 (m, H_α)<br>1.90 (m, H_β)	38.10
5	25.3	CH_2	2.01 (m, H_β)<br>1.90 (m, H_α)	25.09
6	127.4	CH	5.26 (dd, ^3J = 11.0, ^3J = 3.8, 1H)	127.02
7	138.4	C_q	–	139.00
8	40.9	CH_2	2.15 (m, H_α)<br>2.07 (m, H_β)	40.76
9	41.6	CH_2	2.21 (m, H_β)<br>1.55 (m, H_α)	41.38
10	44.9	CH	2.36 (d, J = 10.6, 1H)	44.72
11	48.9	C_q	–	48.79
12	42.0	CH_2	1.54 (m, H_β)<br>1.47 (m, H_α)	41.88
13	41.6	CH_2	1.35 (m, 2H)	41.40
14	68.7	CH	1.35 (m, 1H)	68.52
15	43.2	C_q	–	43.21
16	32.2	CH_3	1.03 (s, 3H)	32.18
17	24.7	CH_3	0.90 (s, 3H)	24.61
18	31.5	CH_3	1.26 (s, 3H)	31.55
19	16.3	CH_3	1.39 (s, 3H)	16.28
20	21.5	CH_3	1.57 (d, J = 1.3, 3H)	21.59

[a] Carbon numbering as shown in Figure S11. [b] Chemical shifts δ in ppm, multiplicity: s = singlet, d = doublet, m = multiplet, coupling constants J are given in Hertz. [c] ^{13}C data in $CDCl_3$ from reference [14] for comparison.

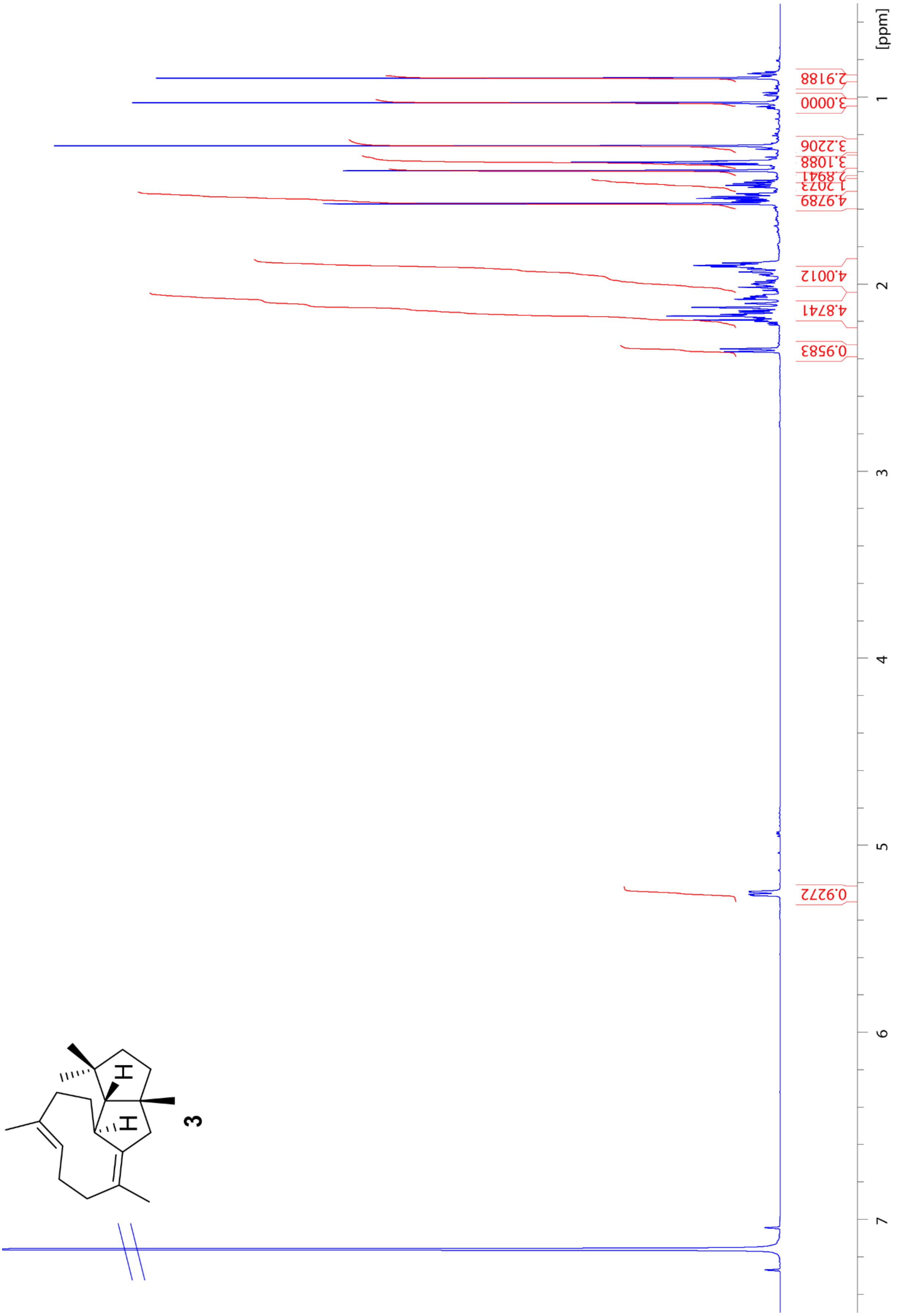

Figure S12. ^{1}H-NMR spectrum of **3** (700 MHz, C_6D_6).

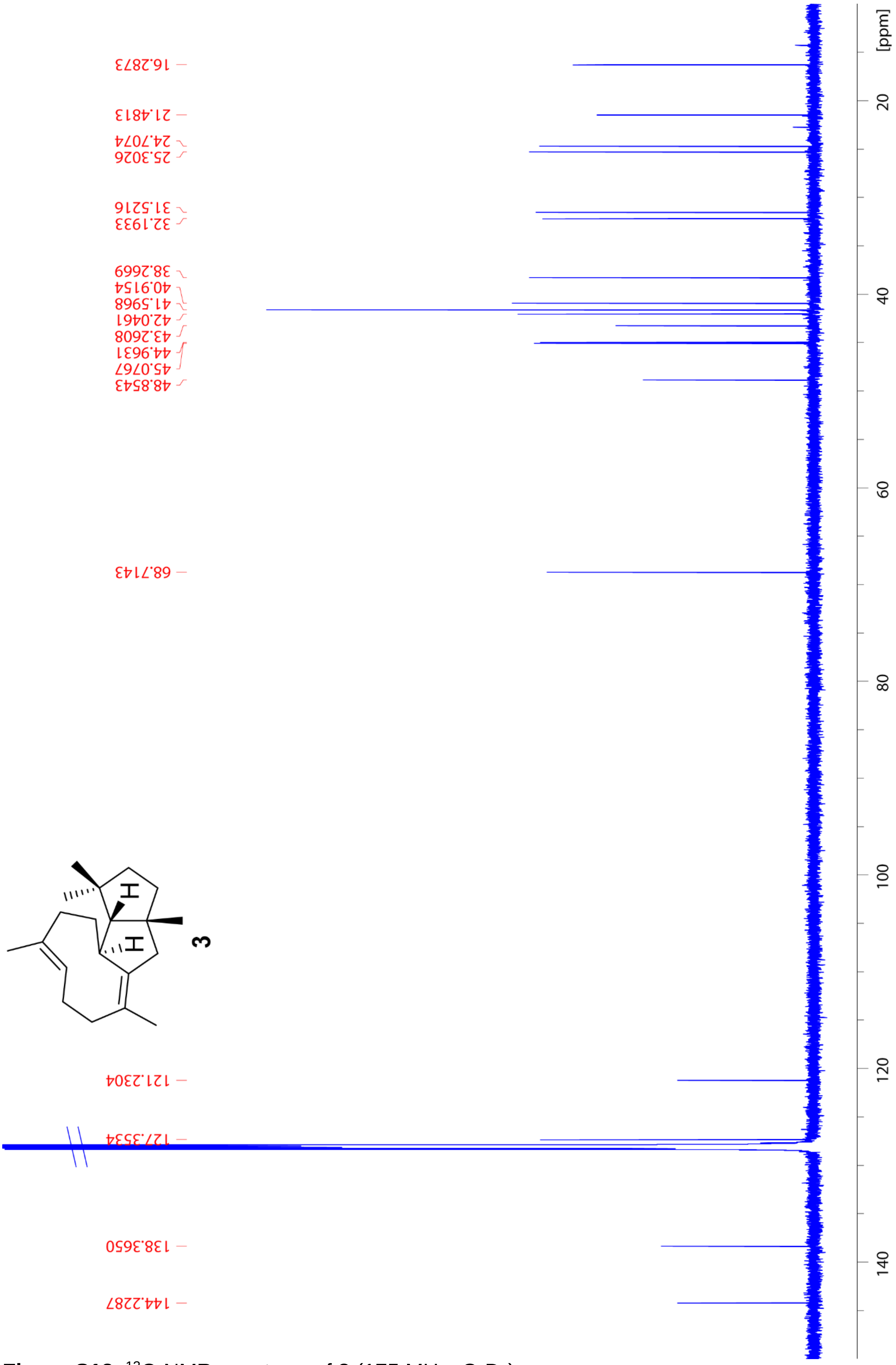

Figure S13. ^{13}C-NMR spectrum of **3** (175 MHz, C_6D_6).

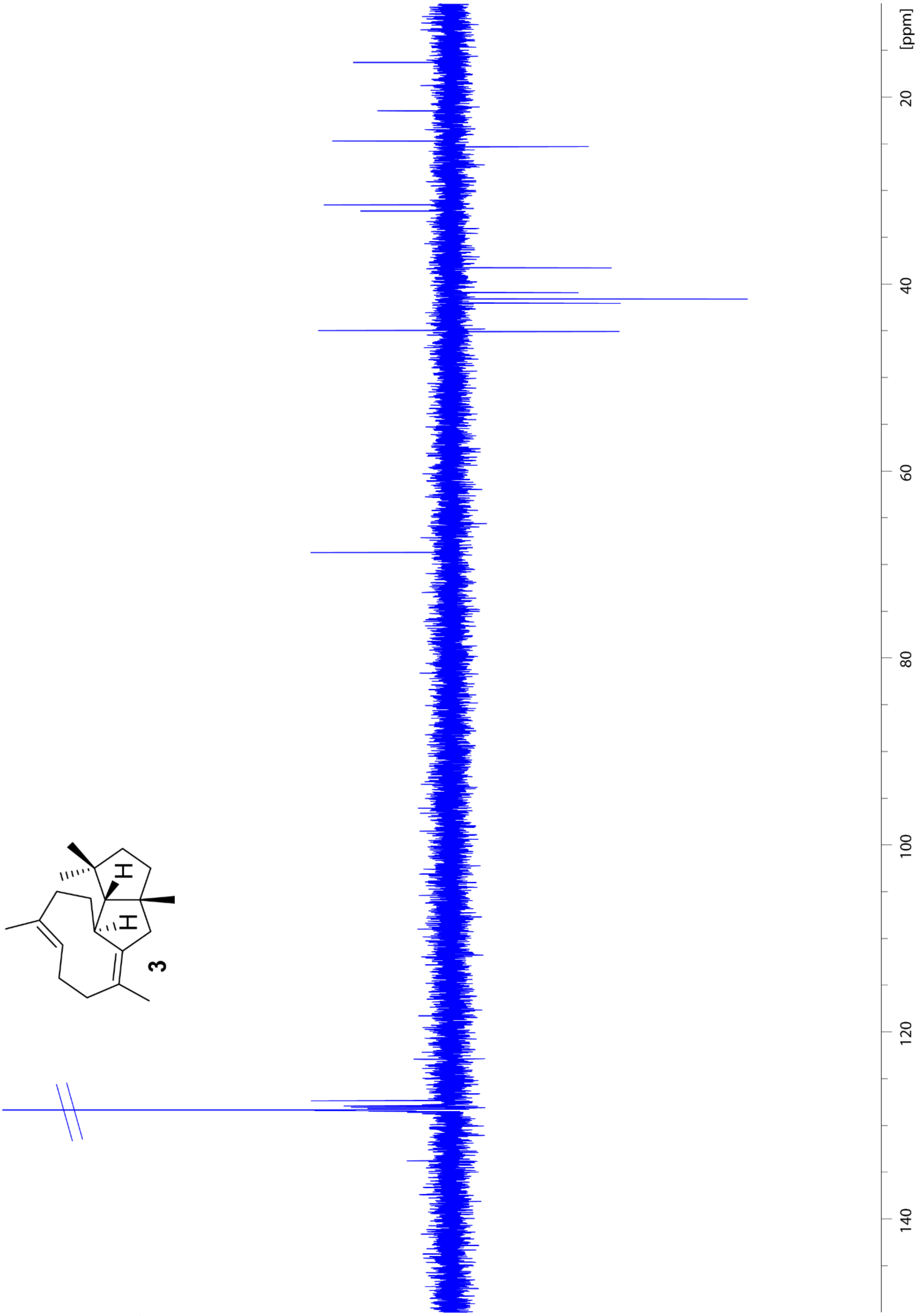

Figure S14. ^{13}C-DEPT 135 spectrum of **3** (175 MHz, C_6D_6).

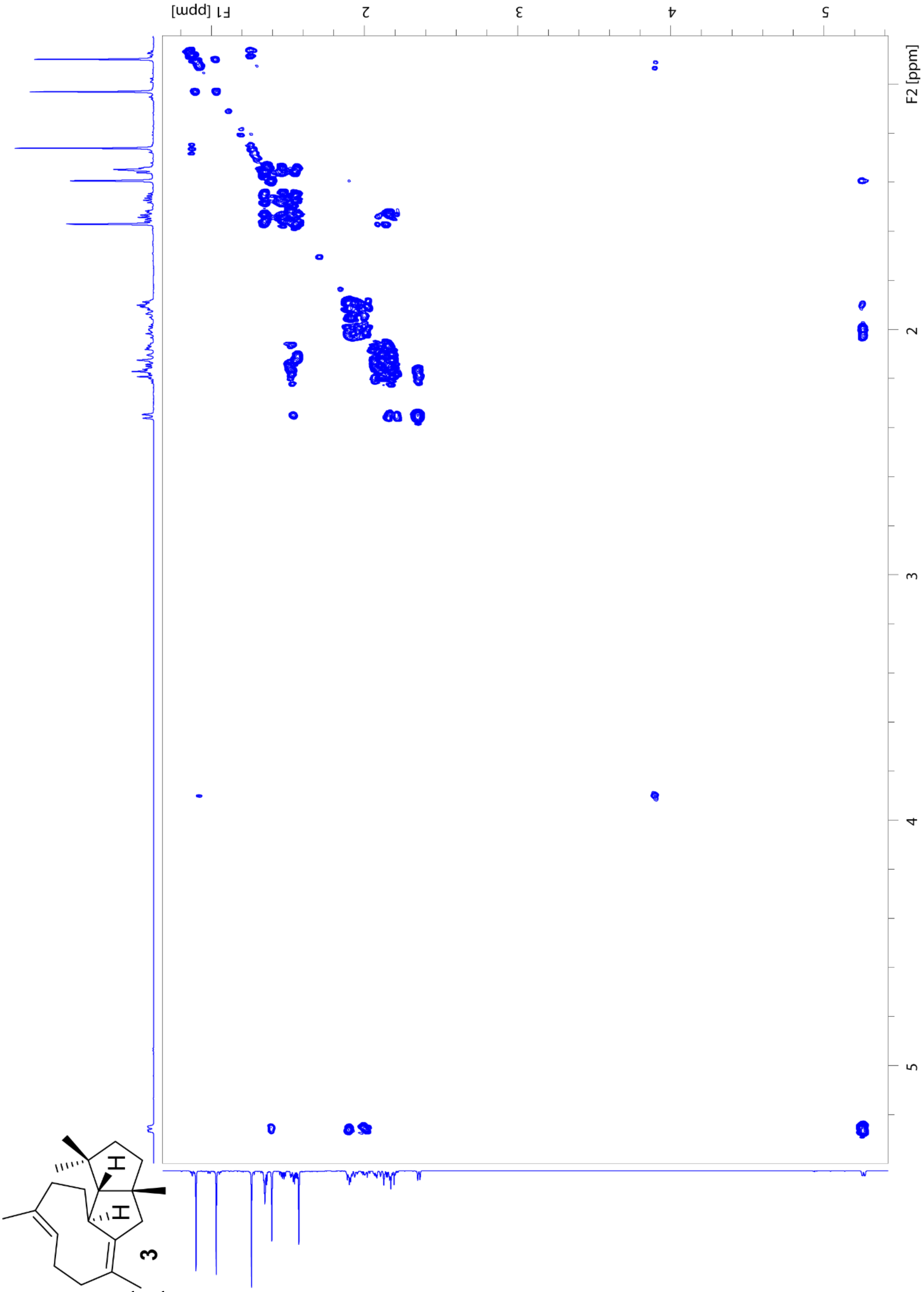

Figure S15. ^{1}H,^{1}H-COSY spectrum of **3** (700 MHz, C_6D_6).

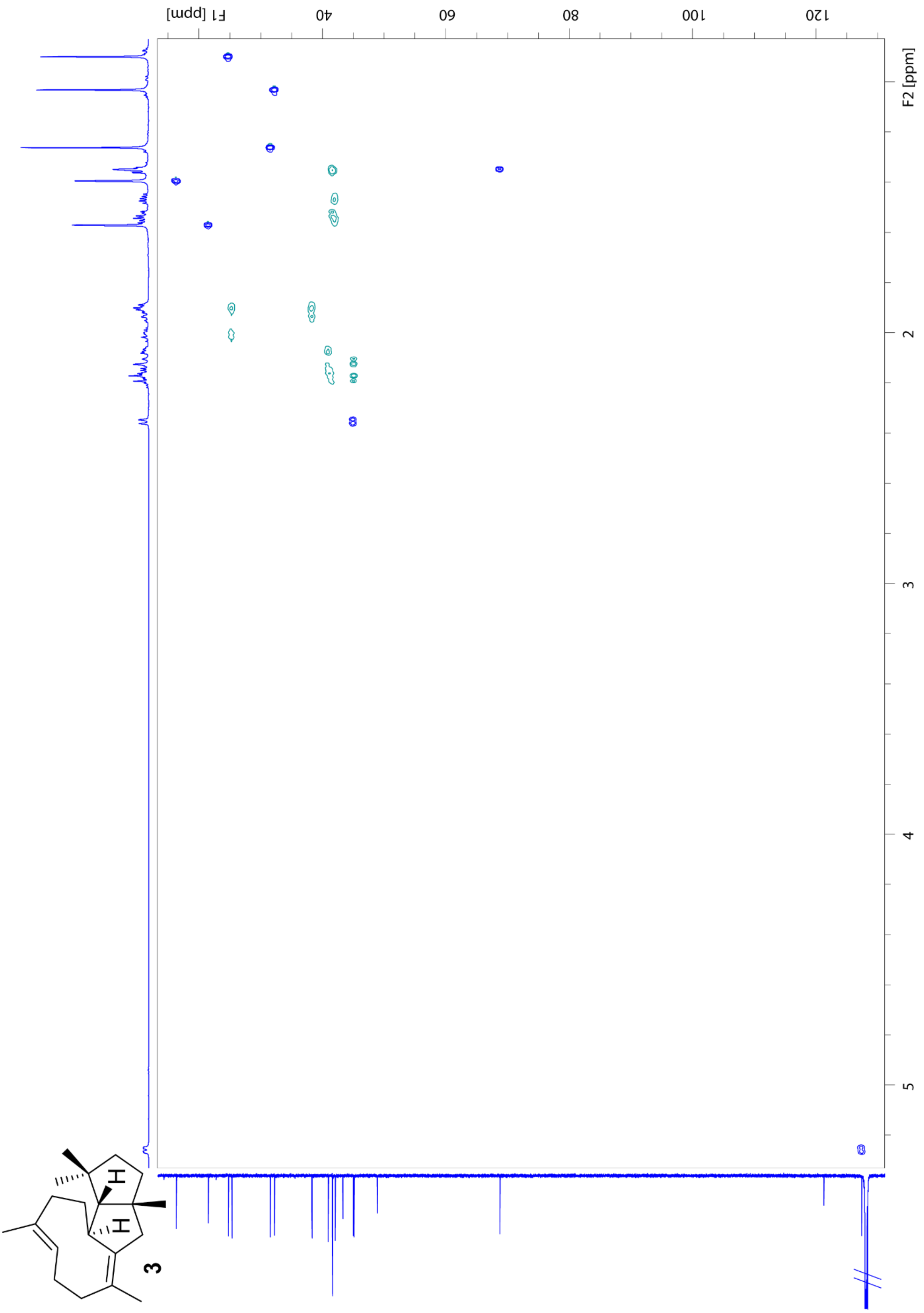

Figure S16. HSQC spectrum of **3** (700 MHz, C_6D_6).

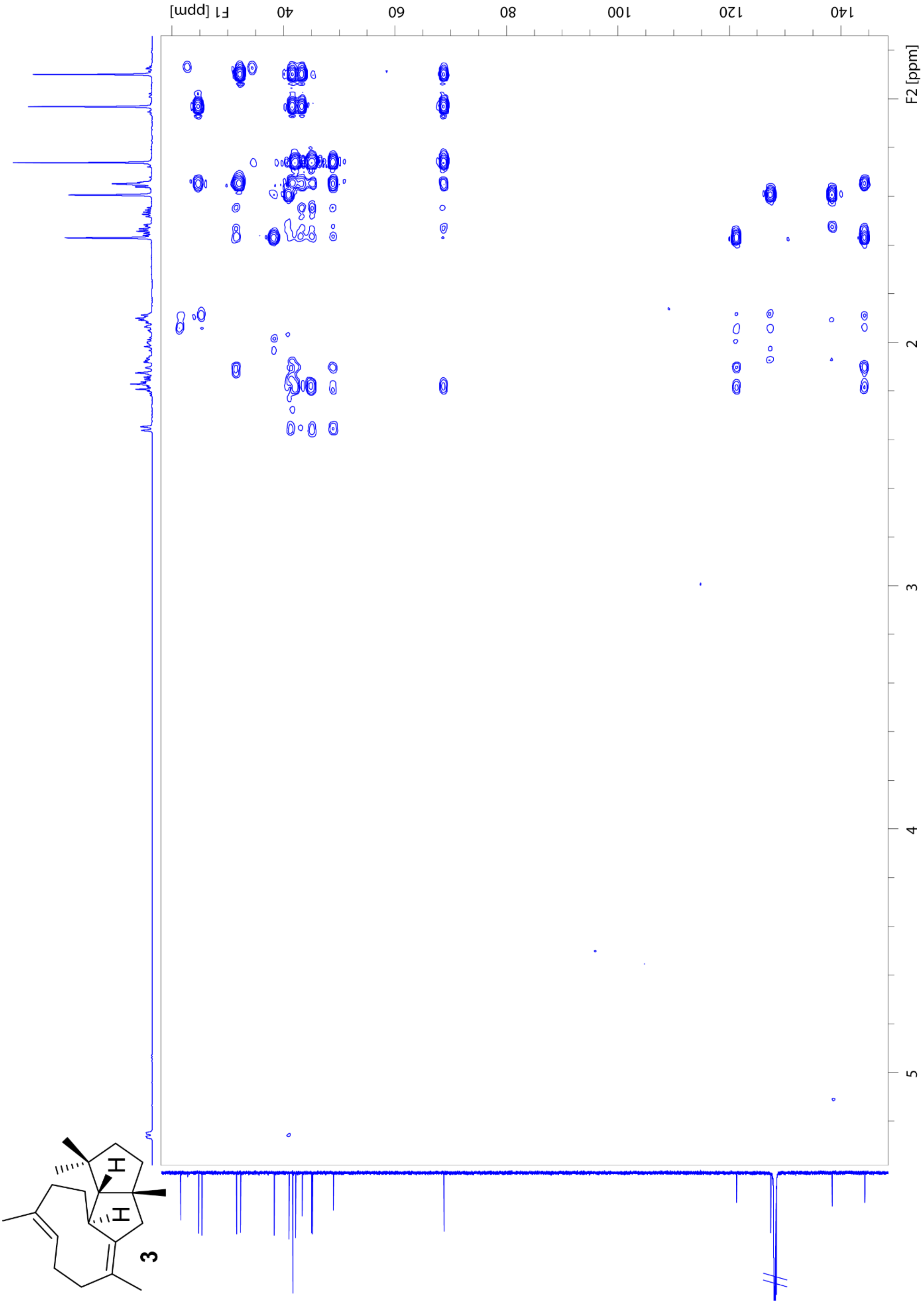

Figure S17. HMBC spectrum of **3** (700 MHz, C_6D_6).

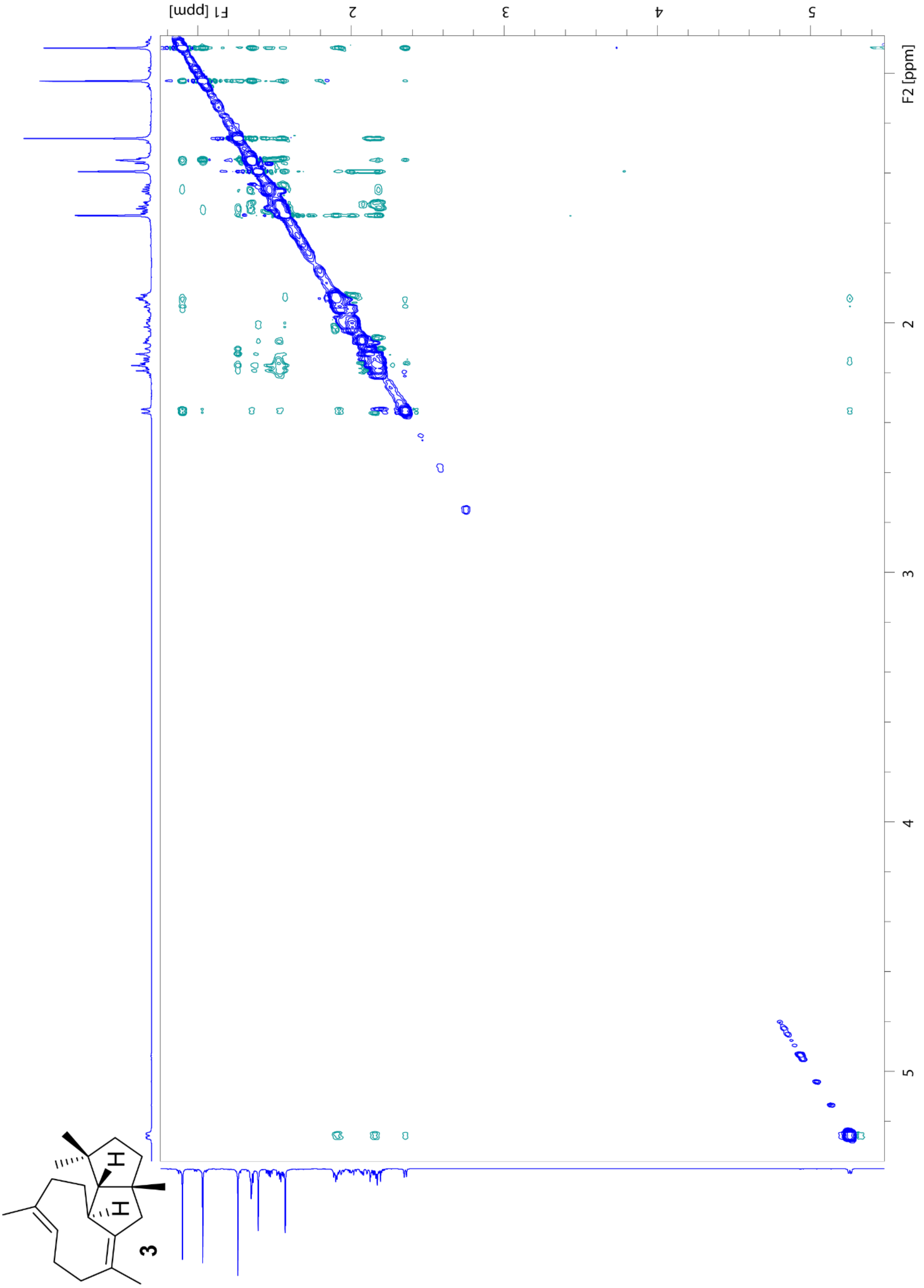

Figure S18. NOESY spectrum of **3** (700 MHz, C_6D_6).

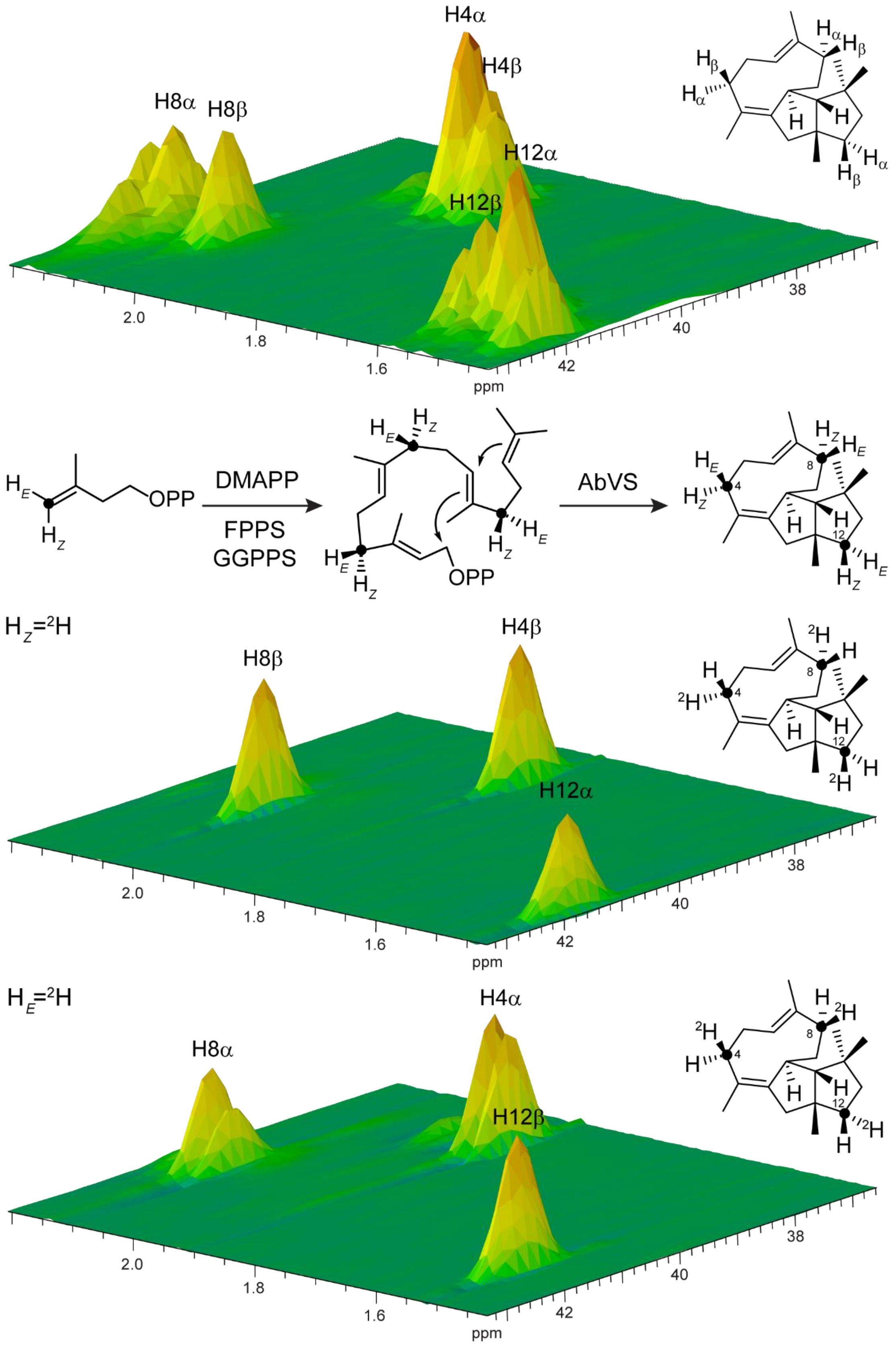

Figure S19. Determination of the absolute configuration of **3** by isotopic labelling experiments. Partial HSQC spectra of unlabelled **3** (top) in comparison to extracts from incubation experiments using AbVS, FPPS, GGPPS and (*Z*)-(4-^{13}C,4-^{2}H)IPP (middle) or (*E*)-(4-^{13}C,4-^{2}H)IPP (bottom) showing the incorporation of deuterium into the expected positions for the shown absolute configuration of **3**. For missing reliable NOE correlations (cf. Figure S11), C8 cannot be used to correlate the absolute configuration. Instead, its orientation can be determined from these experiments. Black dots represent ^{13}C-labelled atoms.

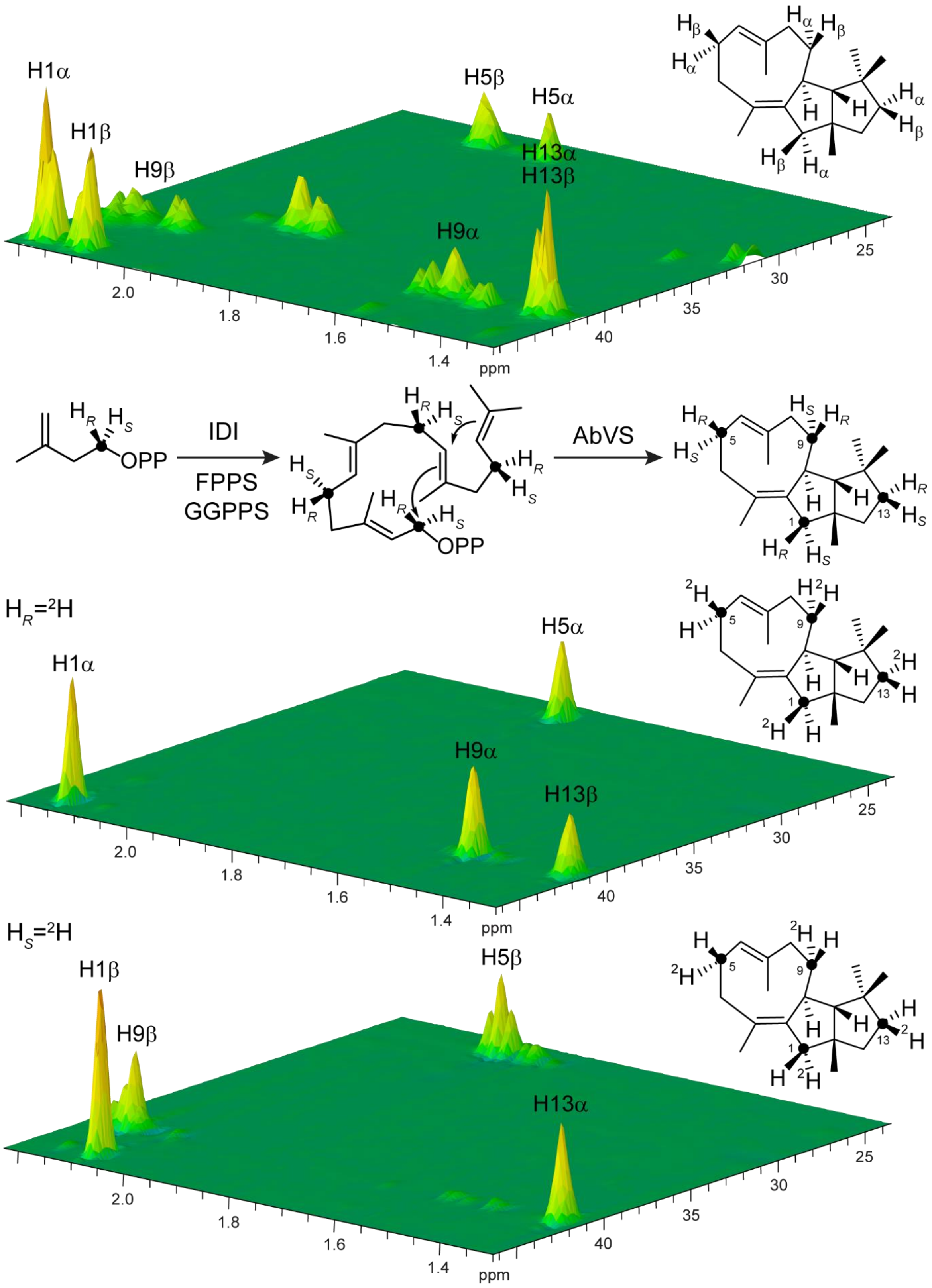

Figure S20. Determination of the absolute configuration of **3** by isotopic labelling experiments. Partial HSQC spectra of unlabelled **3** (top) in comparison to extracts from incubation experiments using AbVS, IDI, FPPS, GGPPS and (*R*)-(1-^{13}C,1-^{2}H)IPP (middle) or (*S*)-(1-^{13}C,1-^{2}H)IPP (bottom) showing deuterium incorporation into the expected positions for the shown absolute configuration of **3**. For missing reliable NOE correlations (cf. Figure S11), C5, C9 and C13 cannot be used to correlate the absolute configuration. Instead, the orientation of C5 and C9 can be determined from these experiments, whereas the signals for H13$_{\alpha}$ and H13$_{\beta}$ are overlapping. Black dots represent ^{13}C-labelled atoms.

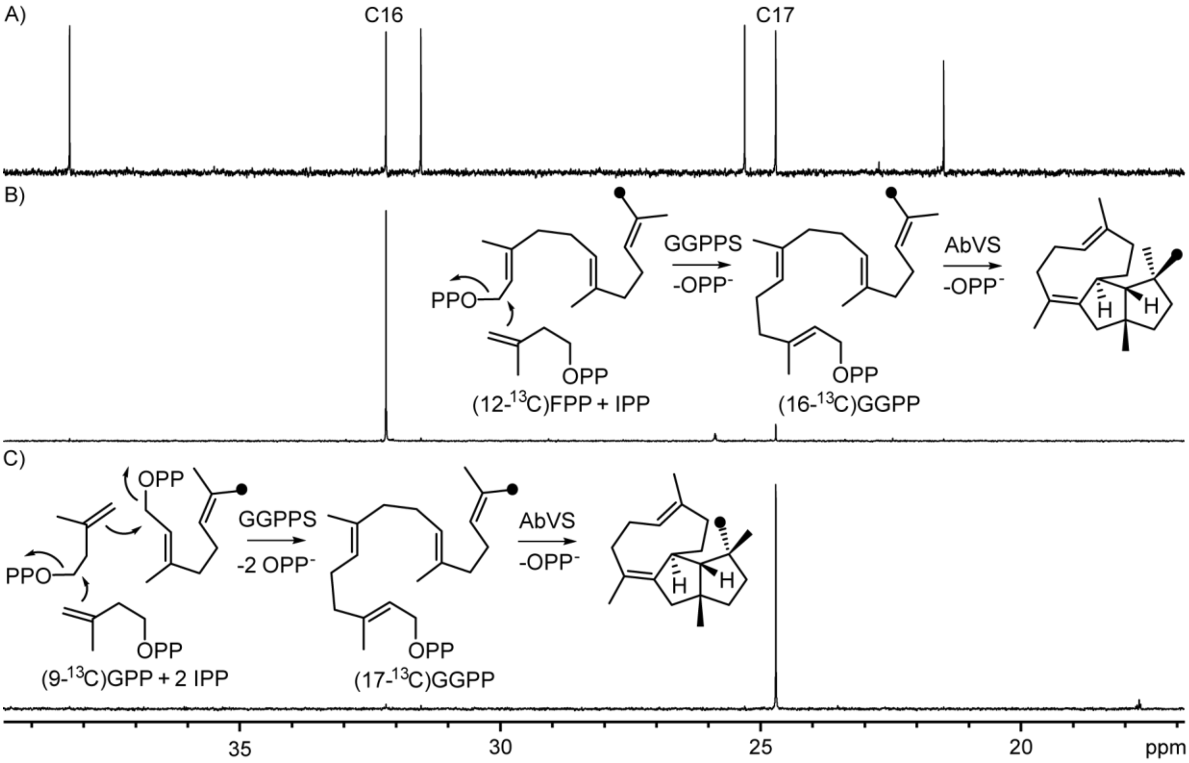

Figure S21. Stereochemical assignment of the geminal methyl groups of **3**. Partial ^{13}C-NMR spectra of A) unlabelled **3**, B) a C_6D_6 extract from the incubation of (12-^{13}C)FPP with IPP, GGPPS and AbVS and C) a C_6D_6 extract from the incubation of (9-^{13}C)GPP with IPP, GGPPS and AbVS. The minor peak visible in B) for C17 arises from partial labelling of C13 in synthetic (12-^{13}C)FPP. Black dots represent ^{13}C-labelled atoms.

GC/MS analyses

A 7890B GC – 5977A mass detector system (Agilent, Santa Clara, CA, USA) was used for GC/MS analyses. The GC was equipped with a HP5-MS fused silica capillary column (30 m, 0.25 mm i. d., 0.50 μm film). GC parameters were 1) inlet pressure: 77.1 kPa, He at 23.3 mL min^{-1}, 2) injection volume: 2 μL, 3) temperature program: 5 min at 50 °C increasing at 5 °C min^{-1} to 320 °C, 4) 60 s valve time, and 5) carrier gas: He at 1.2 mL min^{-1}. MS parameters were 1) source: 230 °C, 2) transfer line: 250 °C, 3) quadrupole: 150 °C and 4) electron energy: 70 eV. Retention indices (*I*) were determined in comparison to a homologous series of *n*-alkanes (C_7-C_{40}).

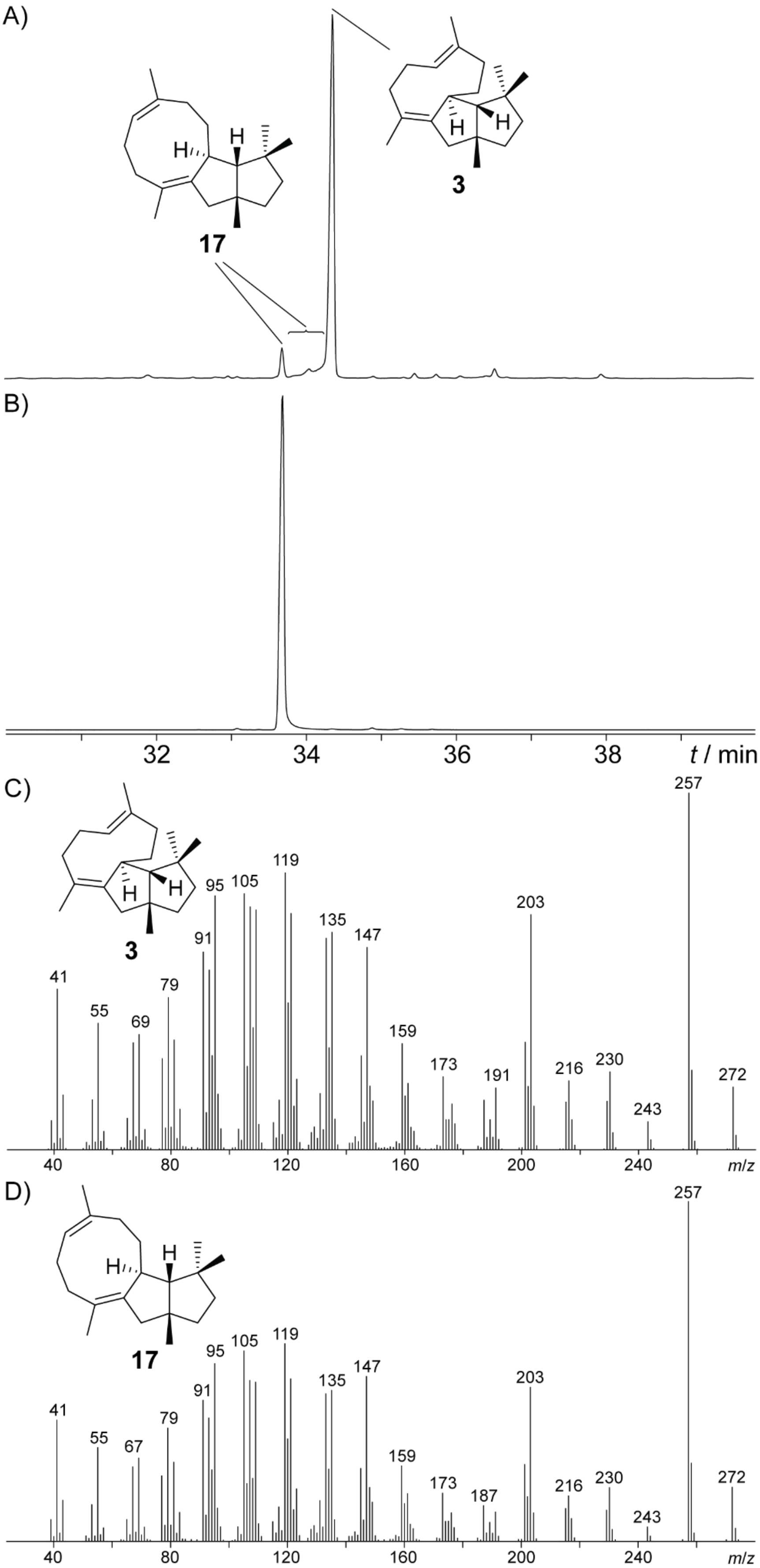

Figure S22. Total ion chromatogram of A) an extract from the incubation of GGPP with AbVS showing thermal degradation of variediene (**3**) occurring both in the injector and on the column, B) purified **17** after thermal isomerisation and EI mass spectra of C) **3** and D) **17**.

Thermal isomerisation of variediene (3)

A solution of (2*Z*,6*E*)-varie-2,6-diene (3, 3.2 mg, 11.8 μmol) in nitrobenzene (0.3 mL) was heated to 210 °C in a 1 mL glass pressure tube (Ace Glass, Vineland, NJ, USA) for 1 h. After cooling to room temperature, the solution was applied to a SiO_2 flash column and chromatographed [pentane] to yield (2*Z*,6*Z*)-varie-2,6-diene (1.4 mg, 5.3 μmol, 45%) as a colourless oil.

(2*Z*,6*Z*)-Varie-2,6-diene, (3a*R*,3b*S*,6*Z*,10*Z*,11a*R*)-3,3,6,10,11a-pentamethyl-2,3,3a,3b,4,5,8,9,11,11a-decahydro-1*H*-cyclonona[*a*]pentalene (17). R_f (pentane) = 0.92. $[\alpha]_D^{20}$ = –61.8 (*c* 0.14, C_6H_6). HRMS (APCI): *m*/*z* = 272.2497 (calc. for $[C_{20}H_{32}]^+$ 272.2499). GC (HP5-MS): *I* = 1787. MS (EI, 70 eV): *m*/*z* (%) = 272 (16), 257 (100), 243 (4), 230 (16), 216 (13), 203 (45), 201 (23), 191 (8), 187 (10), 173 (14), 159 (22), 147 (48), 135 (44), 133 (43), 121 (48), 119 (58), 109 (47), 107 (47), 105 (56), 95 (52), 93 (36), 91 (41), 81 (23), 79 (33), 77 (19), 69 (24), 67 (22), 55 (28), 41 (36), cf. Figure S22. IR (diamond ATR): $\tilde{\nu}$ / cm^{-1} = 3038 (w), 2929 (s), 2860 (s), 1457 (m), 1375 (m), 1172 (w), 1158 (w), 1055 (w), 826 (m), 530 (w), 477 (w). NMR data are given in Table S5 and Figures S23–S30.

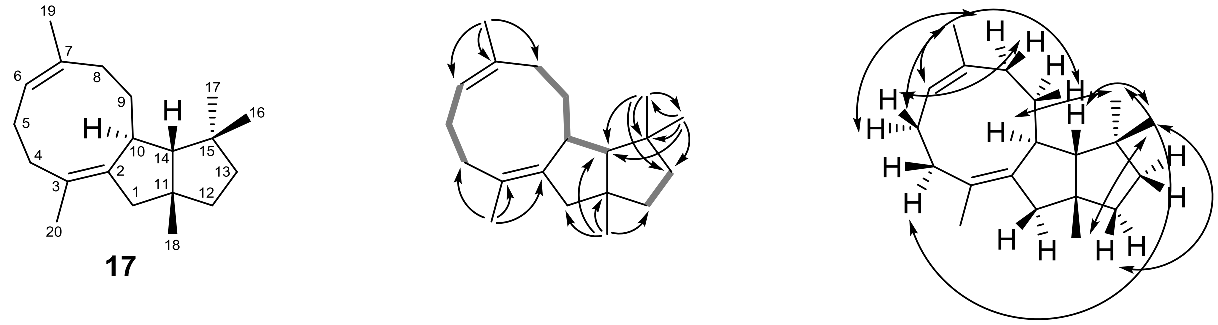

Figure S23. Structure elucidation of (2*Z*,6*Z*)-varie-2,6-diene (**17**). ^{1}H,^{1}H-COSY correlations are shown in bold grey, single headed arrows represent HMBC correlations and NOESY correlations are shown by double headed arrows.

Table S5. NMR spectral data of (2*Z*,6*Z*)-varie-2,6-diene (**17**) in C_6D_6 recorded at 298 K.

C[a]	^{13}C[b]		^{1}H[b]
1	44.43	CH_2	2.23 (d, ^2J = 15.1, H_α)
			2.11 (m, H_β)
2	140.13	C_q	–
3	126.47	C_q	–
4	35.95	CH_2	2.10 (m, H_α)
			1.94 (m, H_β)
5	26.24	CH_2	2.09 (m, H_α)
			1.97 (m, H_β)
6	126.67	CH	5.59 (t, ^3J = 7.7)
7	136.48	C_q	–
8	30.04	CH_2	2.26 (m, H_β)
			1.66 (m, H_α)
9	36.39	CH_2	1.54 (m)
10	43.01	CH	2.49 (dd, ^3J = 10, 7.5)
11	49.00	C_q	–
12	42.00	CH_2	1.55 (m, H_β)
			1.50 (m, H_α)
13	41.82	CH_2	1.38 (m)
14	67.86	CH	1.33 (s)
15	43.25	C_q	–
16	31.56	CH_3	1.02 (s)
17	24.70	CH_3	0.88 (s)
18	32.21	CH_3	1.26 (s)
19	22.70	CH_3	1.71 (s)
20	21.08	CH_3	1.65 (s)

[a] Carbon numbering as shown in Figure S23. [b] Chemical shifts δ in ppm, multiplicity: s = singlet, d = doublet, t = triplet, m = multiplet, coupling constants *J* are given in Hertz.

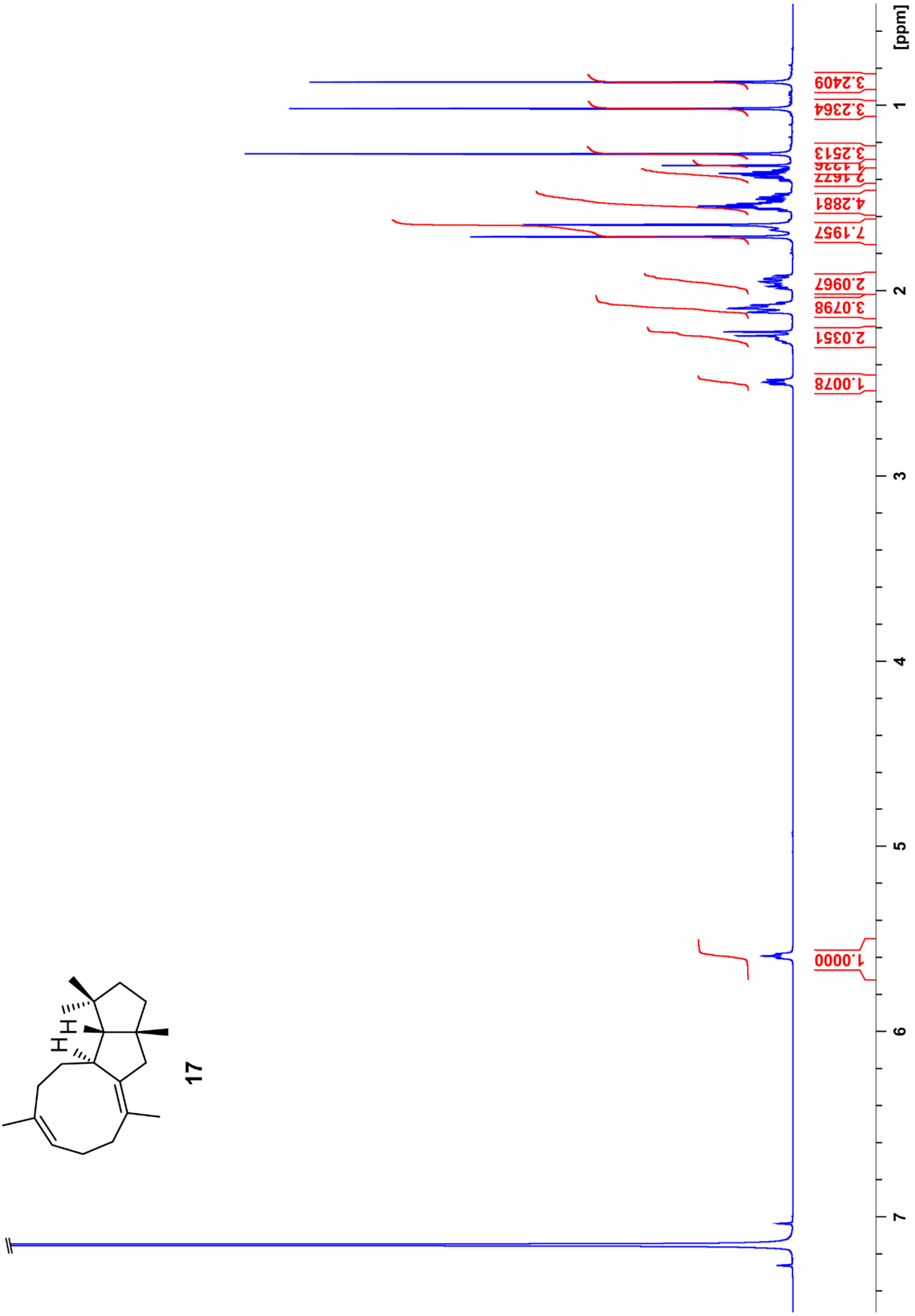

Figure S24. ^{1}H-NMR spectrum of **17** (700 MHz, C_6D_6).

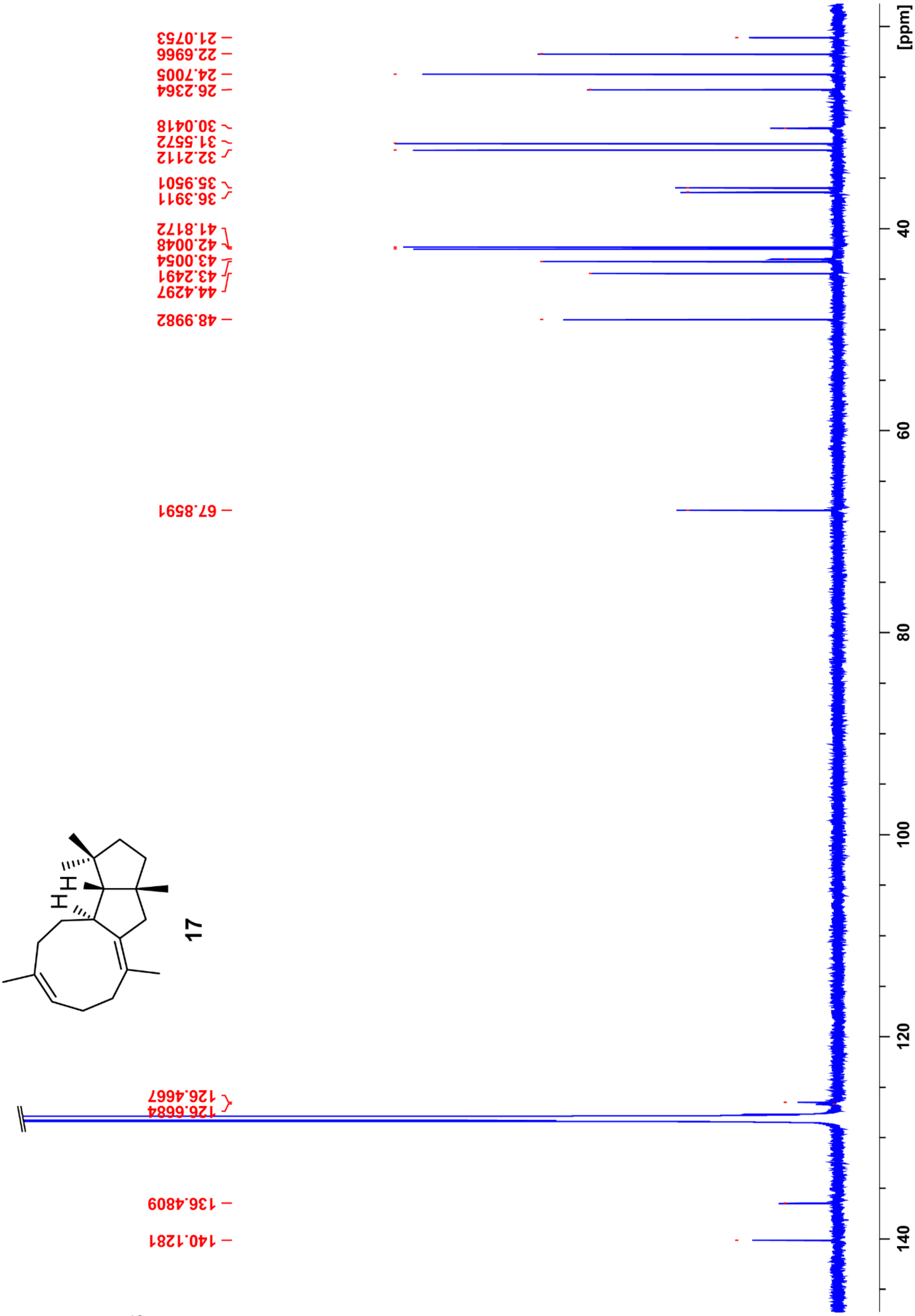

Figure S25. ^{13}C-NMR spectrum of **17** (175 MHz, C_6D_6).

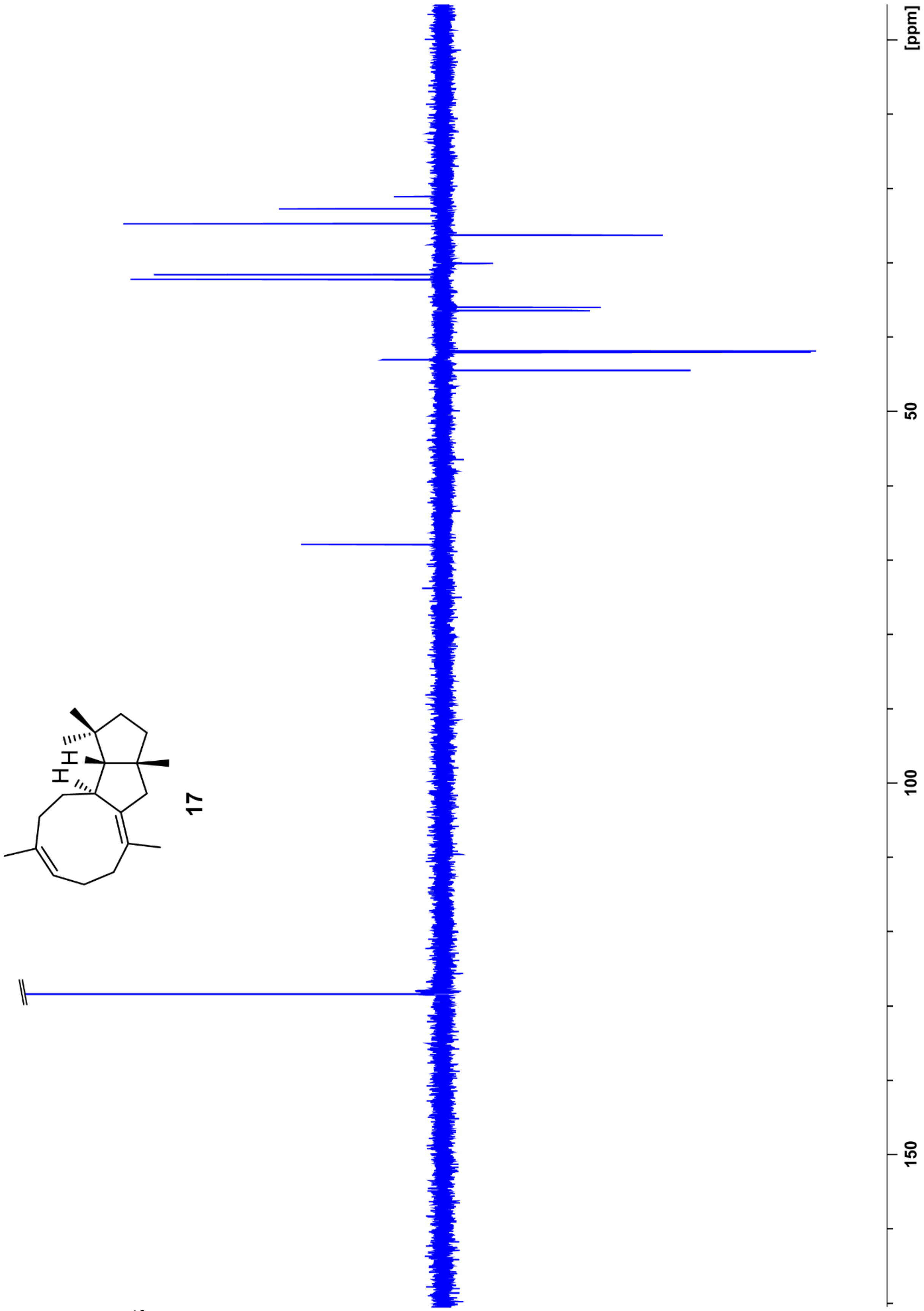

Figure S26. ^{13}C-DEPT 135 spectrum of **17** (175 MHz, C_6D_6).

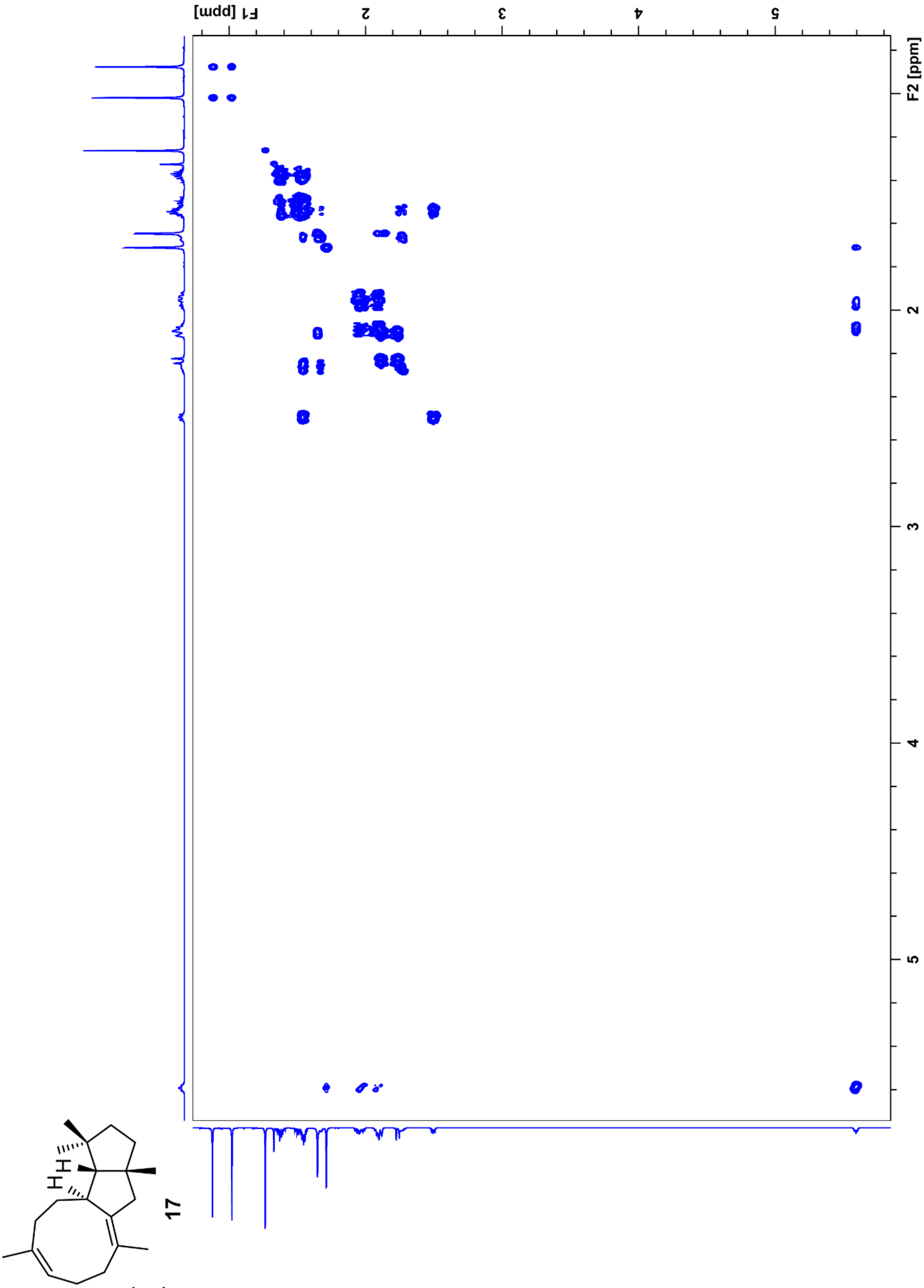

Figure S27. $^{1}H,^{1}H$-COSY spectrum of **17** (C_6D_6).

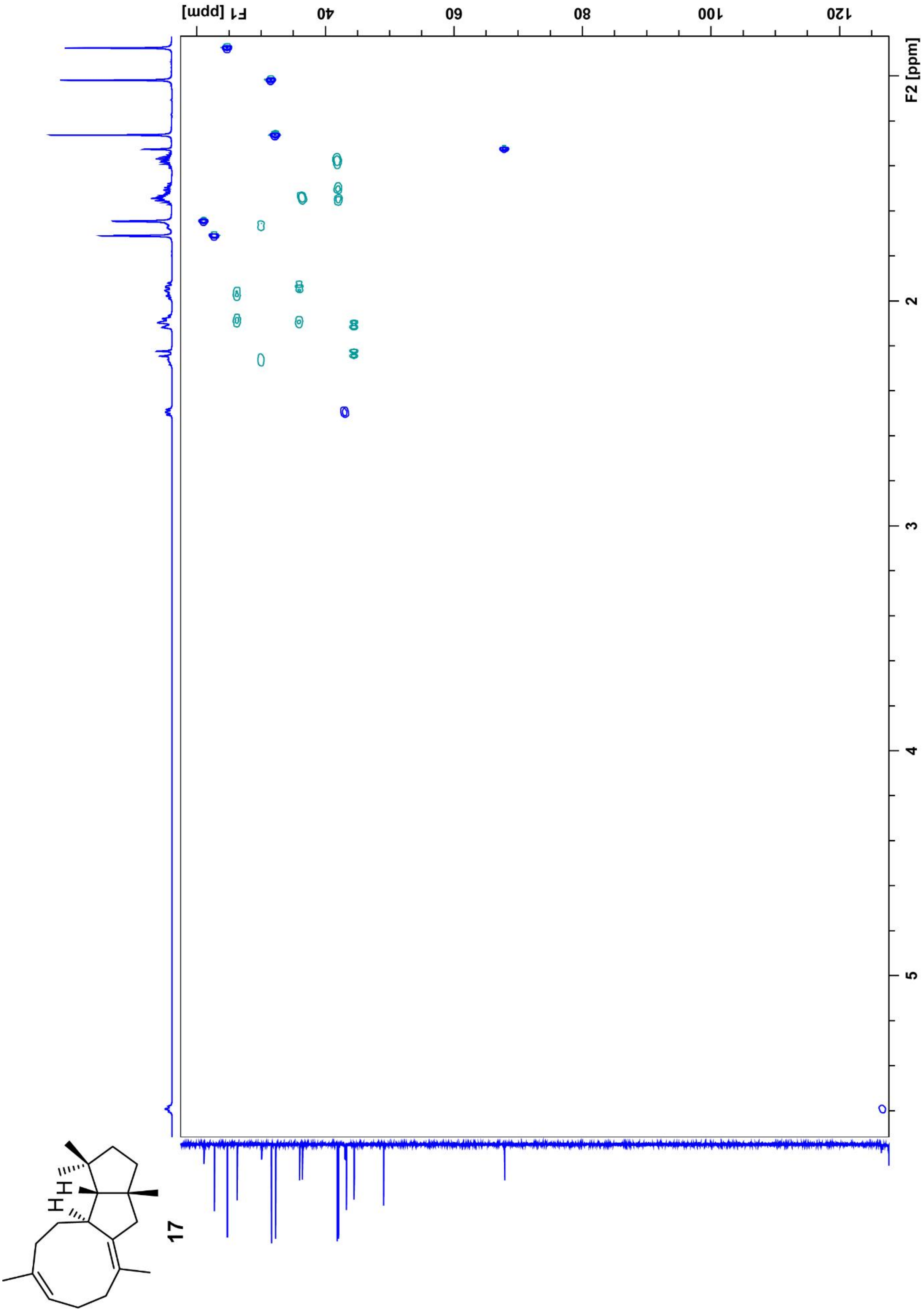

Figure S28. HSQC spectrum of **17** (C_6D_6).

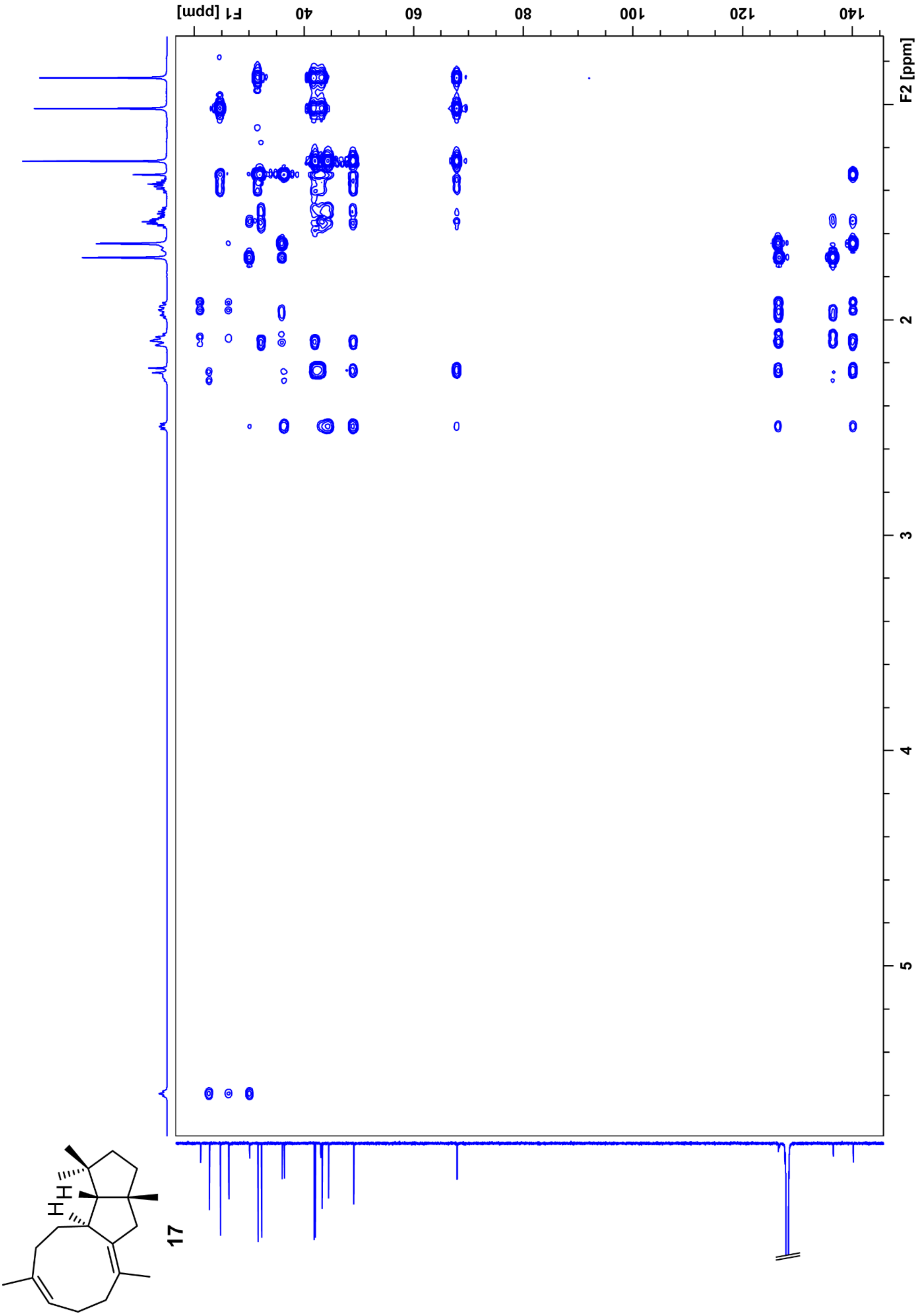

17

Figure S29. HMBC spectrum of **17** (C_6D_6).

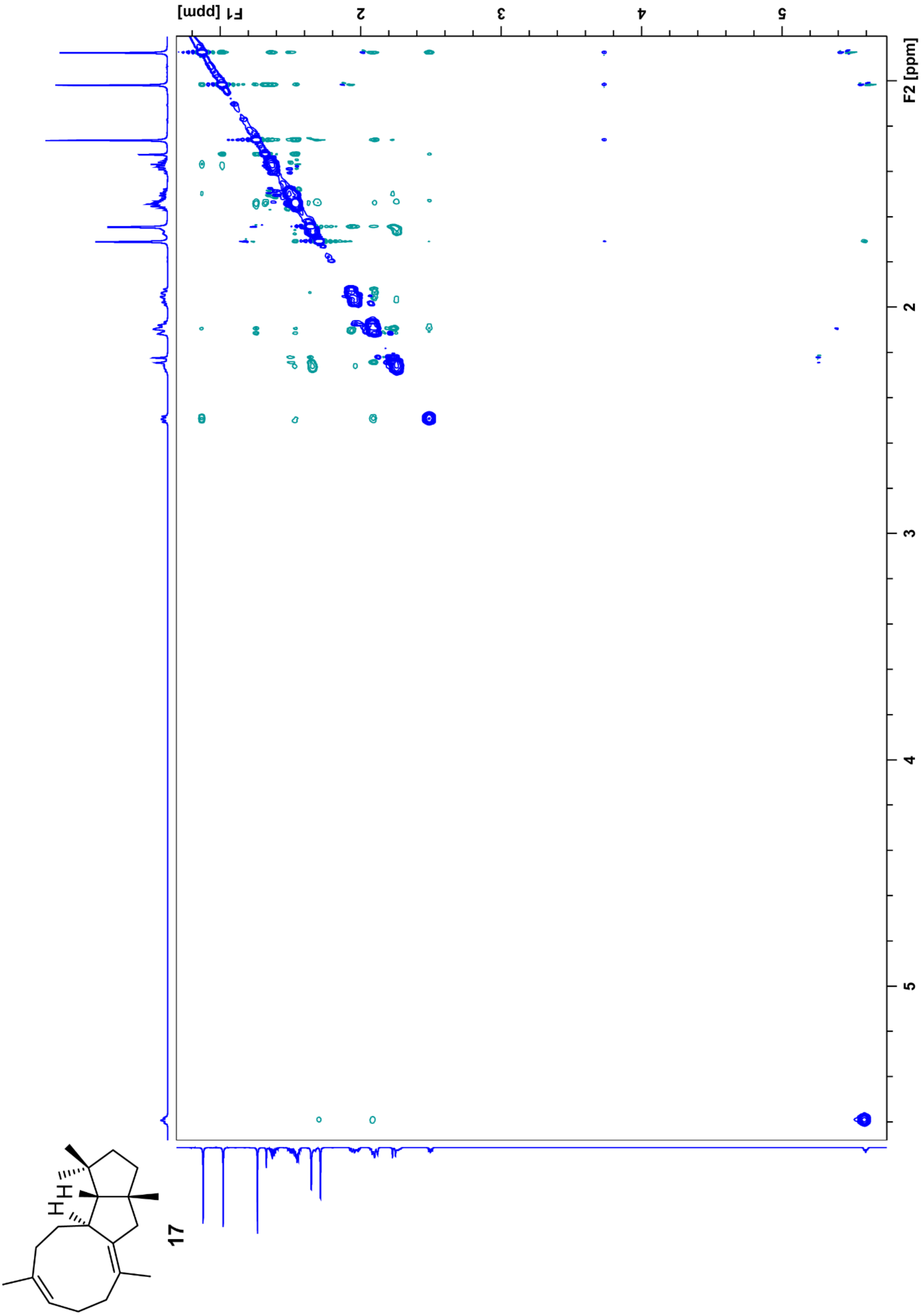

Figure S30. NOESY spectrum of **17** (C_6D_6).

Thermal isomerisation of isotopically labelled samples of variediene (3)

The four isotopically labelled variediene samples, prepared as described above, were concentrated in vacuo and dissolved in nitrobenzene (0.2 mL). After thermal treatment at 210 °C in a 1 mL pressure tube for 1 h, the samples were passed through a glass pipette charged with SiO_2 and a wool-filter by pentane after cooling. The flowthrough was collected shortly before elution of nitrobenzene was observed, the solutions were concentrated under reduced pressure and dissolved in C_6D_6 for NMR measurement.

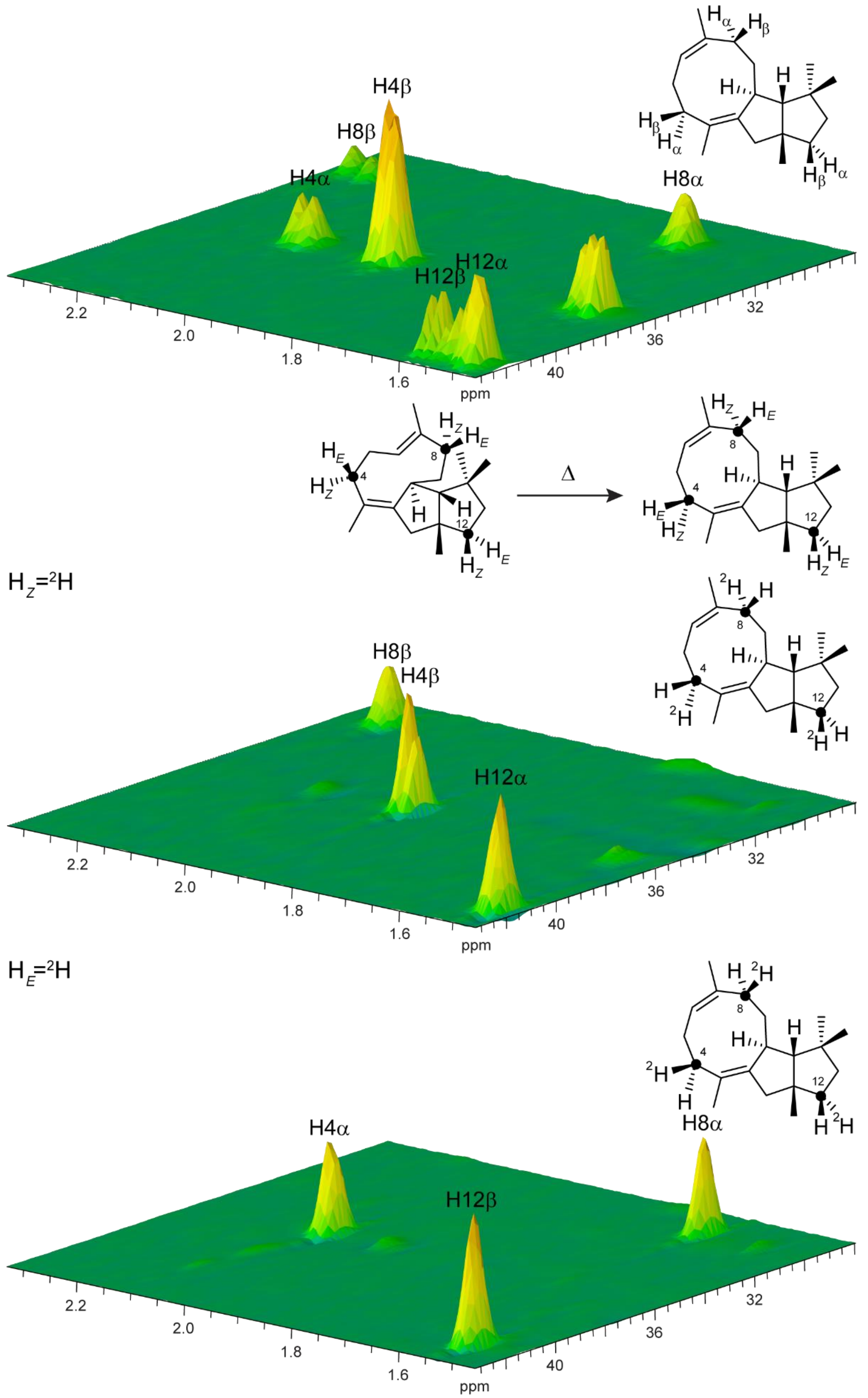

Figure S31. Thermal isomerisation of isotopically labelled variediene (**3**) samples at positions C4, C8 and C12 to **17**. Partial HSQC of unlabelled **17** (top) compared to deuterated H_Z (middle) and deuterated H_E (bottom). The observed incorporations are in line with the methylene configurations observed by NOESY (cf. Figure S23). Black dots represent ^{13}C-labelled carbon atoms.

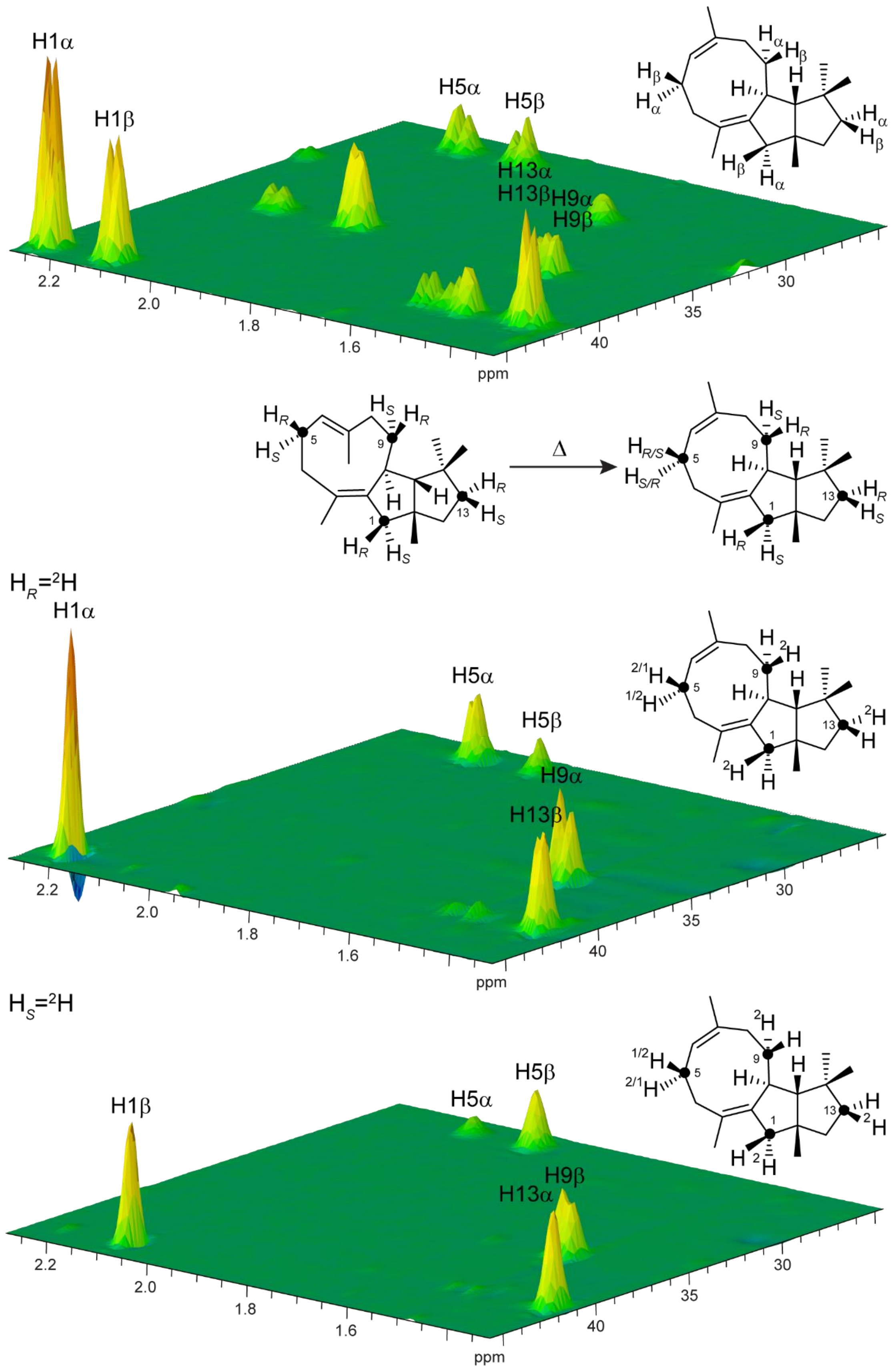

Figure S32. Thermal isomerisation of isotopically labelled variediene (**3**) samples at positions C1, C5, C9 and C13 to **17**. Partial HSQC of unlabelled **17** (top) compared to deuterated H_R (middle) and deuterated H_S (bottom). The observed incorporation for C1 is in line with the methylene configuration observed by NOESY (Figure S23). For C5, a partial epimerisation can be observed after isomerisation, pointing to a role of the corresponding hydrogens in the isomerisation mechanism. Black dots represent ^{13}C-labelled carbon atoms.

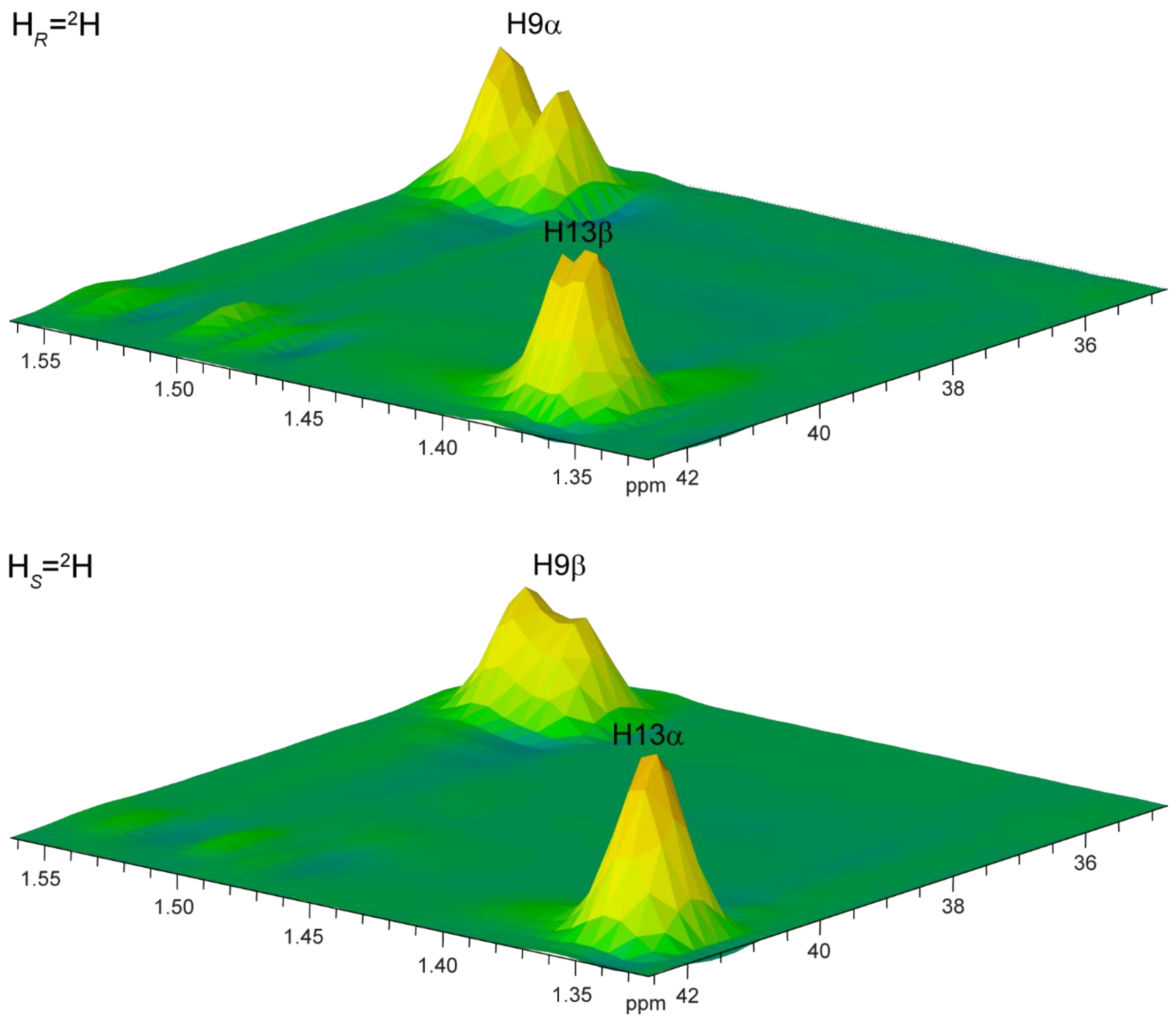

Figure S32 (continued). Enlarged representation of HSQC spectra from thermally isomerised labelled variediene (**3**) samples for C9 and C13. Whereas a selective incorporation is observed for the hydrogen atoms of C13, a conclusion for deuterium scrambling for C9 is not possible because of overlaying signals.

References

[1] G. Bian, Y. Han, A. Hou, Y. Yuan, X. Liu, Z. Deng, T. Liu, *Metab. Eng.* **2017**, *42*, 1.
[2] G. R. Fulmer, A. J. M. Miller, N. H. Sherden, H. E. Gottlieb, A. Nudelman, B. M. Stoltz, J. E. Bercaw, K. I. Goldberg, *Organometallics* **2010**, *29*, 2176.
[3] P. Rabe, J. Rinkel, E. Dolja, T. Schmitz, B. Nubbemeyer, T. H. Luu, J. S. Dickschat, *Angew. Chem. Int. Ed.* **2017**, *56*, 2776.
[4] J. Rinkel, L. Lauterbach, J. S. Dickschat, *Angew. Chem. Int. Ed.* **2019**, *58*, 452.
[5] P. Rabe, L. Barra, J. Rinkel, R. Riclea, C. A. Citron, T. A. Klapschinski, A. Janusko, J. S. Dickschat, *Angew. Chem. Int. Ed.* **2015**, *54*, 13448.
[6] G. Bian, J. Rinkel, Z. Wang, L. Lauterbach, A. Hou, Y. Yuan, Z. Deng, T. Liu, J. S. Dickschat, *Angew. Chem. Int. Ed.* **2018**, *57*, 15887.
[7] T. Mitsuhashi, J. Rinkel, M. Okada, I. Abe, J. S. Dickschat, *Chem. Eur. J.* **2017**, *23*, 10053.
[8] L. Lauterbach, J. Rinkel, J. S. Dickschat, *Angew. Chem. Int. Ed.* **2018**, *57*, 8280
[9] P. Rabe, J. Rinkel, B. Nubbemeyer, T. G. Köllner, F. Chen, J. S. Dickschat, *Angew. Chem. Int. Ed.* **2016**, *55*, 15420.
[10] J. Rinkel, J. S. Dickschat, *Org. Lett.* **2019**, *21*, 2426.
[11] R. D. Giets, R. H. Schiestl, *Nat. Protoc*. **2007**, *2*, 31.
[12] J. S. Dickschat, K. A. K. Pahirulzaman, P. Rabe, T. A. Klapschinski, *ChemBioChem* **2014**, *15*, 810.
[13] M. M. Bradford, *Anal. Biochem.* **1976**, *72*, 248.
[14] B. Qin, Y. Matsuda, T. Mori, M. Okada, Z. Quan, T. Mutsuhashi, T. Wakimoto, I. Abe, *Angew. Chem. Int. Ed.* **2016**, *55*, 1658.
